# A “waste” of time?: the synthesis of a magnesium-substituted aluminophosphate from an unconventional source of magnesium†

**DOI:** 10.1039/d5dt00694e

**Published:** 2025-05-19

**Authors:** Catherine H. Crockett, Jessica R. Bedward, Phoebe K. Clayton, John S. O. Evans, H. Christopher Greenwell, Russell A. Taylor

**Affiliations:** a Department of Chemistry, Durham University Durham DH1 3LE UK russell.taylor@durham.ac.uk; b Department of Earth Sciences, Durham University Durham DH1 3LE UK

## Abstract

Magnesium-substituted microporous aluminophosphates (MgAlPOs) are tuneable framework materials that exhibit strong Brønsted acidity and have found great utility as catalysts for a wide variety of green transformations. Typically, refined magnesium salts, such as magnesium acetate, are the source of magnesium used in the synthesis of MgAlPOs. However, magnesium is classified as a critical raw material, thus finding alternative sources of magnesium remains an important endeavour. Here we demonstrate the use of struvite, a mineral which can form as a problematic waste material, as a viable source of magnesium for the synthesis of MgAlPO-5 (AFI framework). Exploration of synthesis conditions showed that using struvite over magnesium acetate as the Mg source led to increased chabazite (CHA) contamination when triethylamine was employed as the template. The CHA phase impurity could be reduced through synthesis optimisation, though not eliminated when struvite was utilised. Phase pure AFI from struvite and magnesium acetate was formed when *N*,*N*-methyldicyclohexylamine was used as a template, and the gel stirred during crystallisation. Electron microscopy revealed that the struvite derived MgAlPO-5 materials exhibit a distinct surface morphology featuring nanorod-like growths, presumably associated with the magnesium source, although the underlying cause remains unclear. Despite this we show that both materials are acidic (DRIFTS and CD_3_CN adsorption studies) and are active catalysts for the dehydration of ethanol to ethylene in the gas phase under flow conditions. In contrast, unsubstituted AlPO-5 shows limited acidity and negligible catalytic activity, validating the incorporation of magnesium into the framework from both magnesium sources. TEM-EDS maps also support this finding, showing a homogenous distribtution of Al, P and O (and Mg) for all materials synthesised. This work demonstrates that struvite can be used in MgAlPO synthesis, under optimised conditions, showing that repurposing this unconventional magnesium source is far from “a waste of time”.

## Abstract 2

A synthetic analogue of a waste material, struvite, is valorised by being utilised as a magnesium source in the synthesis of magnesium-substituted aluminophosphates.

## Graphical abstract

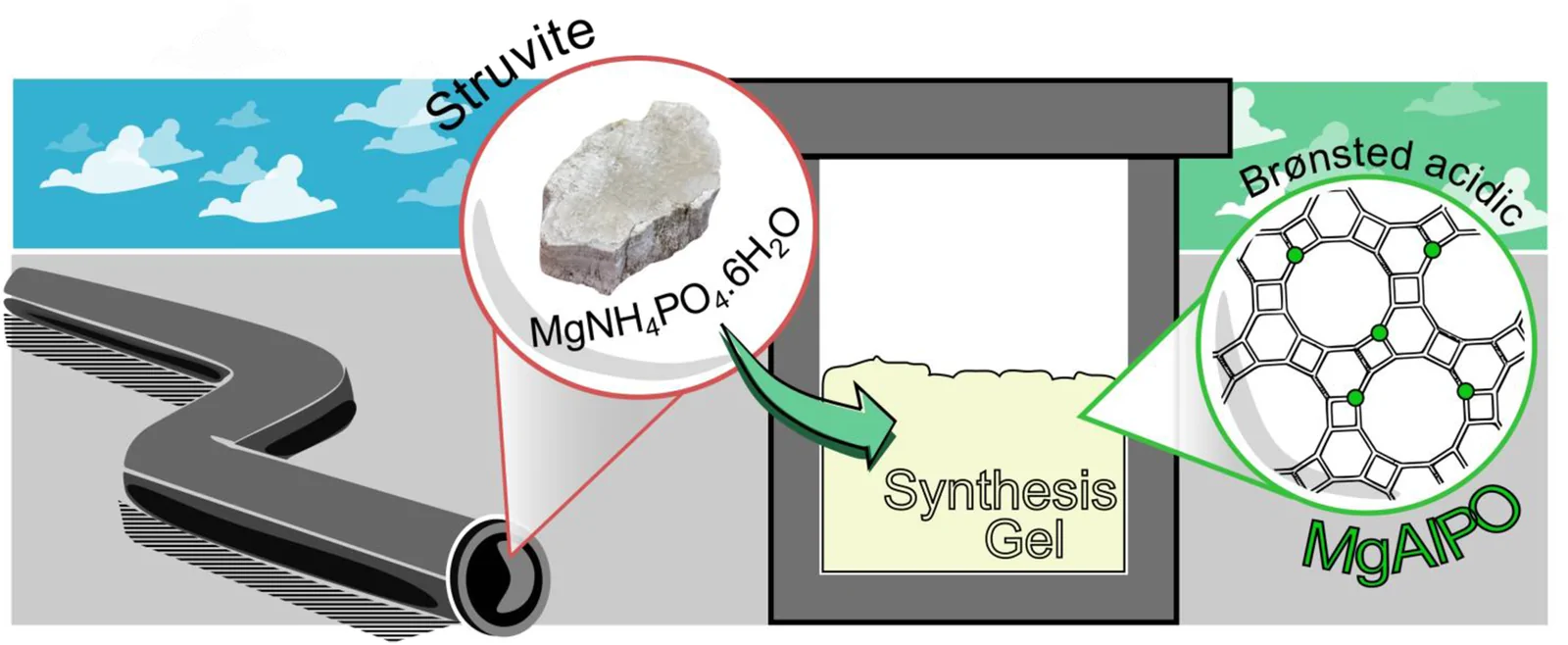

## 1. Introduction

Microporous aluminophosphates (AlPOs), first described in the seminal 1982 report,^1^ are oxide framework materials, comprising strictly alternating [AlO_4_]^−^ and [PO_4_]^+^ tetrahedra, resulting in a neutral framework.^2–4^ These building units can form a plethora of different frameworks, each with varying ring sizes and channel dimensionality, similar to their zeolite counterparts.^2,4^ The target framework of this work is AFI (AlPO-5) which consists of 12, 6 and 4 membered rings, and unidimensional channels (Fig. 1).^2^ The diameter of the largest ring opening is 7.3 Å.^5^

**Fig. 1:**
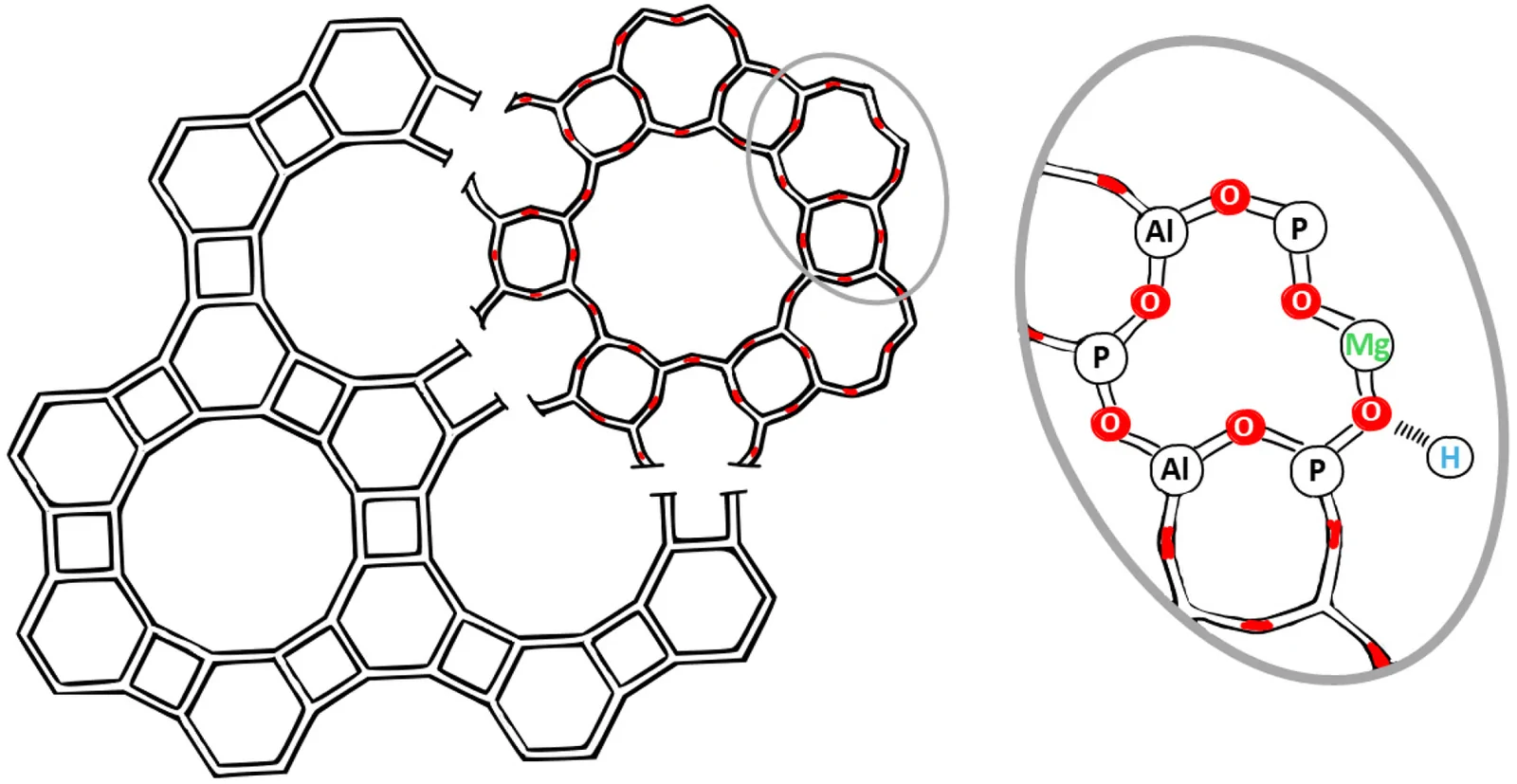
LHS: A diagram of the AFI framework showing the framework based on tetrahedral (T) atoms, and with oxygen (upper right quarter). RHS: a magnified segment showing the effect of magnesium substitution on the framework and subsequent creation of Brønsted acidity.

To induce different properties into these neutral framework materials, the framework aluminium can be substituted for divalent cations, or the phosphorus for tetravalent cations.^3,4,6,7^ These substitutions create a charge imbalance on the framework, which gives the material Brønsted acidity when compensated by a proton (Fig. 1).^7^ Elements commonly substituted into the AlPO framework include Mg, Si, Fe, Ni, Cu, Zn, Zr, Cr, Mn and Ti.^8–10^ The synthesis can be facile, often achieved by adding an appropriate metal salt to the initial synthesis gel.

In their Brønsted acidic form substituted AlPOs are catalytically useful materials and show great potential as solid acid catalysts.^4,11–14^ This potential has been realised in the commercial deployment of a silicon substituted AlPO (SAPO-34) for the methanol to olefins (MTO) process.^15^ However, understanding how changes to the heteroatom substitution and framework type affect the methanol to hydrocarbon (MTH) reaction remains a very active field of research.^16–19^ The wide tuneability of substituted AlPOs for MTH chemistry is exemplified in their use as co-catalysts in the synthesis of value-added hydrocarbons directly from CO_2_,^18,20–23^ and hence these catalysts will be important in the transition to a green energy landscape.^24,25^ Furthermore, substituted AlPOs show great promise as catalysts for the upgrading of bio-based feedstocks such as lignocellulose and lignocellulose derivatives,^26^ including bioethanol to ethylene,^27^ demonstrating the wide versatility of substituted AlPO catalysts for the sustainable production of chemicals.

In this work, magnesium is utilised as the divalent cation to replace aluminium, forming MgAlPOs. These materials find great utility as Brønsted acid catalysts (Table S2†), but the synthesis of the magnesium-substituted frameworks is dominated by using refined and unsustainable magnesium salts as the metal precursor (Table S1†). To avoid the use of primary chemical feedstocks and minimise pressure on a diminishing magnesium supply (listed as a critical raw material by the EU since 2014),^28,29^ we have devised a more sustainable synthesis method for these catalytically important materials. The new synthesis uses a Mg-containing waste material, struvite, to replace a conventional and less sustainable magnesium salt.

Struvite, MgNH_4_PO_4_·6H_2_O, is a mineral, that precipitates out of solution in concentrated, nutrient-rich waste streams.^30^ Such waste streams can be found at wastewater treatment plants, and on landfill sites.^31^ Struvite is sparingly soluble (p*K*_sp_ = 9.4–13.26)^32^ so deposits collect and can lead to pipe blockages.^33^ Solubility increases at pH values below 5^34^ so deposits can be removed with acid washing, or manual cleaning.^35^ Alternatively, struvite formation can be prevented by complexing the phosphate with ferric or aluminium salts.^35^ Another approach is to recover struvite, using existing technology^30,32^ to facilitate this process, however without further uses the cost of this would not be justified. Struvite does possess the qualities of a slow-release fertiliser, making it a sustainable alternative to conventional products.^36^ However, the use of struvite from wastewater sources as a fertiliser has stalled due to concerns over microbiological safety.^37,38^

Synthetic struvite forms orthorhombic, coffin-like crystals (Fig. S1†). The crystal structure contains [PO_4_]^3−^ tetrahedra, distorted [Mg(H_2_O)_6_]^2+^ octahedra and [NH_4_]^+^ groups.^39,40^ Struvite will form when the component ions are present in concentrations nearing, or above supersaturation, which has a strong dependency on solution pH.^32^ For example, the time taken for struvite precipitation decreased from days to hours when the solution pH increased from 7 to 8.5 respectively.^32^ In a wastewater setting the removal of carbonic acid in the form of CO_2_ is promoted in areas of high turbulence (bends within pipework or pumps)^41^ creating a higher pH environment. These turbulent areas will therefore be more susceptible to struvite fouling than others.^42^ The presence of other ions in the matrix will also affect struvite precipitation and often result in the formation of mixed phases. The presence of calcium can lead to the formation of calcium phosphate for example.^43^ There is also some evidence to suggest that struvite formation can occur biologically, in the presence of microorganisms, as well as chemically.^44^

Struvite recovery has the potential to be a sustainable method for recycling of Mg and P (the latter of which is also classified as a critical raw material).^28^ Therefore, it would be beneficial from economic and environmental perspectives to find a use for this waste material.^45^ Other waste materials have been used in the synthesis of AlPOs previously, the primary example of which is the repurposing of aluminium dross.^46–50^ Another example is the precipitation of a ZnAlPO from phosphatising wastewater containing zinc, a waste product from metal polishing and coasting industries.^51^

In this work we show for the first time, that synthetic struvite can be used as a Mg source for MgAlPO-5. The struvite derived MgAlPO-5 has similar properties to MgAlPO-5 synthesised from a conventional Mg source. This work shows that an unconventional Mg source can be used to create MgAlPOs, adding to ways in which valuable resources can be recovered from wastewater.^52–54^

## 2. Experimental

### 2.1. Preparation of materials

Full experimental details are provided in the ESI† however MgAlPO-5 was prepared as previously reported.^55^ Briefly, both D.I. H_2_O and H_3_PO_4_ were added to a beaker and stirred at a speed of 600 RPM at room temperature. After 10 min a portion of Al(OH)_3_·*x*H_2_O (determined by TGA to contain 26 wt% H_2_O) was added. After a further 10 min, a suspension of struvite or a solution of Mg(OAc)_2_ was then added. Lastly, the template, *N*,*N*-methyldicyclohexylamine (MDCHA) was added dropwise with a pipette and stirred for 90 min at 600 RPM. The final molar ratio of the gel was 0.74 Al: 1.50 P: 0.8 MDCHA: 38.5 H_2_O: 0.03 Mg (water from Al(OH)_3_·*x*H_2_O and H_3_PO_4_ are accounted for in the above ratio, see ESI† for reagent masses and amounts). Aluminium isopropoxide was used for the synthesis of AlPO-5 and replaced Al(OH)_3_·*x*H_2_O in the reported method.^55^

The resultant opaque white gel was divided evenly between six Teflon autoclave liners (∼40 mL internal volume). The mass of each was recorded, and the pH measured with a pH meter (ThermoScientific Orion Star A211). A cylindrical stirrer bar was added to each liner, followed by a Teflon lid. The liners were sealed into the autoclaves and placed in a Carbolite Gero forced convection oven at 180 °C on a six-position stirrer plate (2mag MIXcontrol 20) set at 600 RPM. After 2 h the autoclaves were removed and cooled on ice for 30 min. The solids were washed twice with 40 mL of D.I. water, at which point the washings reached pH 6, then oven-dried at 80 °C overnight. The mass of each as-synthesised (AS) product isolated at this stage in the synthetic procedure is given in the ESI.†

A portion of the AS product was spread thinly onto a calcination dish and calcined in a static air furnace (Carbolite CWF1100) using the following procedure: 80 °C for 1 h, 120 °C for 1 h followed by 16 h at 550 °C (all ramps at a rate of 2.5 °C min^−1^).

The products will be referred to as MgAlPO-5(OAc) (from magnesium acetate) and MgAlPO-5(S) (from struvite) hereon in. A material of AlPO-5 was synthesised from Al(OH)_3_·*x*H_2_O however this was latterly found to contain magnesium, contaminating the intended AlPO-5. This material will be referred to as (c-Mg)AlPO-5. A second AlPO-5 material was synthesised using a different aluminium source without Mg contamination and will be referred to as AlPO-5 (see Table S3†).

### 2.2. Characterisation of materials

The PXRD patterns of all materials were recorded on a Bruker AXS D8 Advance with a Cu Kα source (*λ* = 1.5406 Å) over a 2*θ* range of 2° to 50° with a step size of 0.02°, in Bragg–Brentano geometry. Rietveld refinement was performed using TOPAS;^56–58^ refinement parameters are provided in the ESI (Table S9†). Elemental analysis was performed on an Agilent 5800 VDV. Materials (∼0.01 g) were dissolved in hydrochloric acid (1 mL, 37%) and D.I. H_2_O (9 mL). CHN analysis was conducted on an Exeter Analytical CE-440 elemental analyser. SEM was performed on a Zeiss Sigma 300 VP, with an accelerating voltage of between 5 and 8 kV. Materials were gold sputter coated to a thickness of 35–40 nm. TEM images were collected on a JEOL 2100F, and elemental maps using an Oxford XMax 65. Thermal analysis was performed on a PerkinElmer TGA 8000. Nitrogen isotherms were collected on a Micromeritics ASAP 2020. Prior to analysis materials were degassed at 350 °C for 10 h. DRIFTS spectra were collected on a Bruker Invenio R with a Harrick Praying Mantis™ high temperature reaction chamber. SSNMR (Solid State Nuclear Magnetic Resonance) measurements were recorded using a Bruker Avance III HD spectrometer.

For catalytic testing, reactions under flow conditions were performed using a HEL FlowCAT flow reactor feeding ethanol *via* an Eldex Optos 1 HPLC pump, as outlined by Raynes *et al.*^59^ All materials were prepared for catalysis by first pressing at 10 tons for 30 s in an Apollo Scientific XRF 32 mm die using a Specac hydraulic press. The pressed catalysts were then sieved between 40 to 60 mesh (420–250 μm). Catalyst beds were packed into a stainless-steel reactor with a 4 mm internal diameter and consisted of a 1.3 g SiC pre-bed, followed by 0.3 g of the desired catalyst diluted with 1.2 g SiC and 0.75 g SiC post-bed. All catalysts were pre-treated under flowing N_2_ (40 mL min^−1^) at room temperature for 15 minutes. The temperature in the reactor bed was then increased to 150 °C and 200 °C, and held at each respective temperature for 1.5 h. The ramping rate for each stage was 10 °C min^−1^. Once the desired reaction temperature was reached, ethanol was flowed (0.01 mmol min^−1^) through the reactor bed, fed *via* the HPLC pump. On-line product analysis was performed by GC-MS-BID (Shimadzu GC-2010 Plus) equipped with an RTX-VMS (Thames Restek) fused silica capillary column for mass spectrometry (MS) detection and a ShinCarbon ST (Thames Restek) column for barrier ionization discharge (BID) detection.

## 3. Results and discussion

### 3.1. A note on the synthetic challenges of a MgAlPO-5(S)

Synthesis of MgAlPO-5 from struvite was initially explored using triethylamine (TEA) as the template, which has been widely reported, in combination with Mg(OAc)_2_ as the Mg source, to produce AFI.^60–63^ Even though the magnesium reactant is only a minor component of the overall aluminophosphate gel, it was found to greatly affect the phase purity of the final MgAlPO product. It is well known that chabazite (CHA) co-crystallises with AFI when using TEA as the template, however specific gel ratios and synthesis conditions can yield pure AFI.^55,61,64,65^ Here, we found that conditions that produced pure AFI when using Mg(OAc)_2_ as the Mg-source did not do so when the source was replaced with struvite during initial trials with TEA (full results in Table S4 and Fig. S2†).

Stirring speed, oven time, dissolution of struvite prior to addition to the gel, and oven temperature increases were all investigated prior to changing the template from TEA to MDCHA. The greatest reduction in CHA contamination when using TEA and struvite was observed when stirring at 1200 rpm in the oven (double the speed required to obtain pure AFI from Mg(OAc)_2_ and TEA). The reduced susceptibility to synthetic conditions (and fortuitously, Mg-source) resulted in continuation with MDCHA, which, as reported by Sanchez-Sanchez *et al.*, does yield AFI with high specificity.^55^

We would like to highlight that autoclave stirring in the oven appeared to be particularly important; even a synthesis with Mg(OAc)_2_ and TEA showed AFI/CHA co-crystallisation when not stirred in the oven. Valuable investigations into the synthesis of MgAlPO-5 using synthetic struvite, detailed hereon in, can now be used to inform the future use of waste struvite in the synthesis of these materials, and for others wanting to investigate unconventional and sustainable reactants.

### 3.2. Bulk structure and elemental composition

The PXRD patterns (Fig. 2a) for all AlPO-5 and MgAlPO-5 materials are similar and showed no AlPO-34 (CHA framework) or aluminophosphate tridymite impurities, as is often seen when synthesising the AFI framework.^55^ Additionally, the patterns of the materials synthesised here possess no additional peaks when compared to the reference pattern (Fig. 2a), further confirming the absence of impurities. Furthermore, they all showed little change upon calcination; only intensity changes in the (110) and (200) peaks (2*θ* = 12.5° and 15.0° respectively), presumably due to the removal of the template. This same intensity change was also observed by Xu *et al.* in high temperature *in situ* X-ray diffraction experiments upon release of the template between 100 and 300 °C,^66^ and by Dib *et al.* upon calcination of their AFI materials.^67^ This therefore suggests that, despite the differing content and sources of magnesium, the hexagonal phase and long-range order of the material persists. Rietveld refinement of the patterns (space group = *P*6*cc*) also shows an increase in both lattice parameters (*a* and *c*) upon increasing Mg-content, supporting the incorporation of the larger Mg^2+^ into the framework (Fig. S3, S4–S7 for Rietveld plots, and Table S9† for refinement details). The relationship between Mg-content and the *a* lattice parameter scales more linearly than the *c* lattice parameter (*R*^2^ = 0.986, compared to *R*^2^ = 0.665 respectively).

**Fig. 2:**
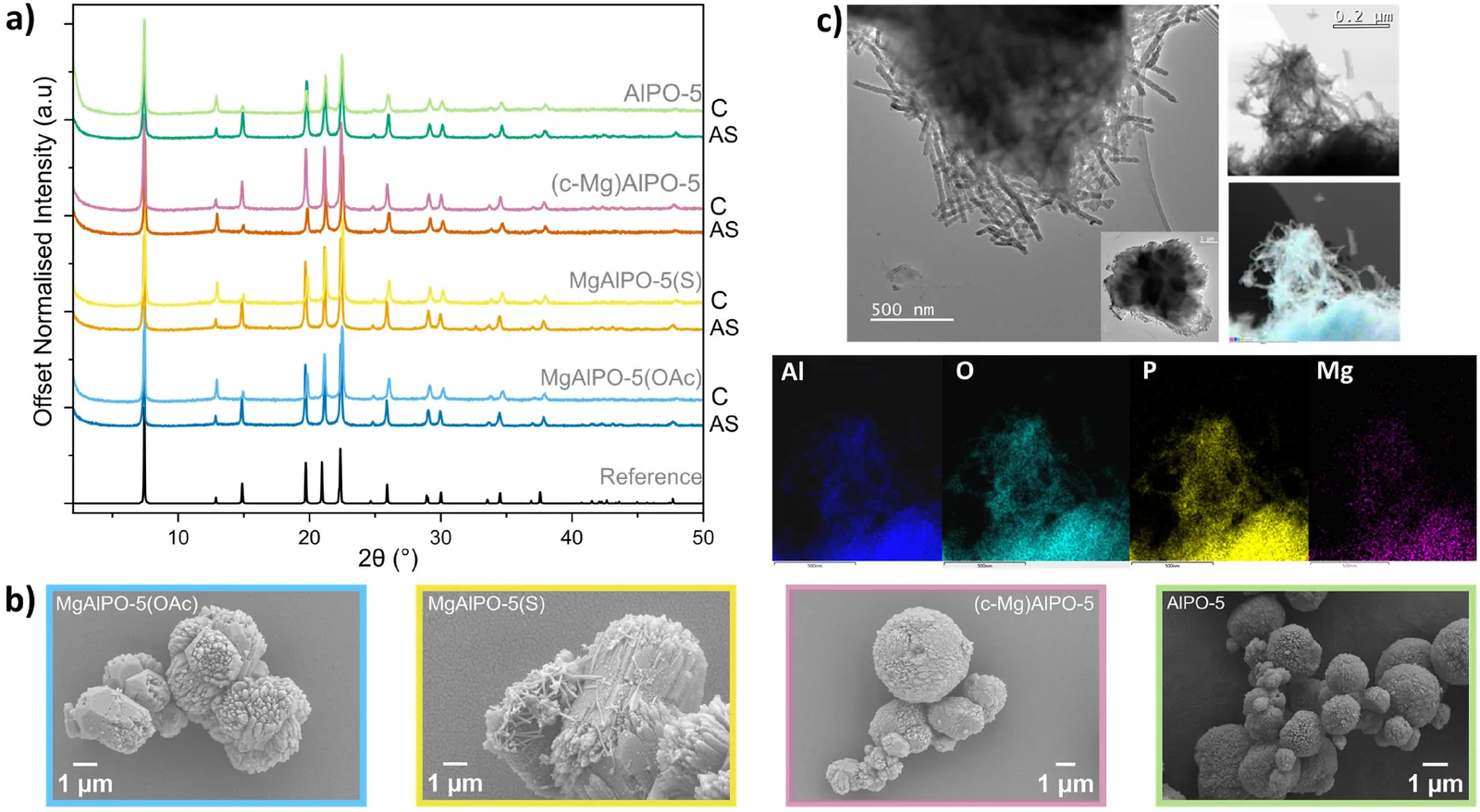
(a) Representative PXRD patterns for all four materials, showing both as-synthesised (AS) and calcined (C) forms, with a reference pattern. All patterns were normalised between 0 and 1 and manually offset for clarity. The AFI reference pattern (tetrapropylammonium fluoride AlPO-5) is from the IZA-SC, originally referenced from Qiu *et al.*;^97^ (b) SEM micrographs of the calcined materials, from left to right, MgAlPO-5(OAc), MgAlPO-5(S), (c-Mg)AlPO-5 and AlPO-5; (c) TEM image of the edge of a calcined MgAlPO-5(S) particle showing the small nano-sized growths, with brightfield image and chemical map showing Al, O, P and Mg.

Elemental analysis indicates that incorporation of magnesium, throughout the bulk of all Mg-substituted materials, was similar. The ratio (Mg + Al/P) remains between 0.940–1.040 (±0.003–0.015) for all materials (Fig. S8†). Following elemental analysis it became apparent that the commercial Al(OH)_3_·*x*H_2_O contained magnesium, thereby contaminating the intended AlPO-5 material (labelled (c-Mg)AlPO-5). Given these results, elemental data was used to ascertain the contribution of the intended magnesium source, and that from the aluminium source to the total Mg incorporation. The total %Mg incorporation in MgAlPO-5(OAc) and MgAlPO-5(S) was 88% and 71% respectively. Since the total %Mg incorporation into the final MgAlPO-5 (in both cases) exceeded the amount from each individual source, both must have contributed. However, the specific contribution from each source cannot be definitively determined (Fig. S9†).

After calcination, CHN analysis reveals that all materials have extremely low amounts of C and N present, suggesting the successful removal of the MDCHA template (Fig. S10†). Hydrogen content is significantly higher in the materials containing magnesium. This increase is hydrogen is likely due to higher levels of intra-porous water arising from the increased hydrophilicity of the Mg-substituted materials. The increased water content is supported by both TGA (section 3.5) and ^27^Al SSNMR (section 3.4) analysis.

### 3.3. Morphology and porosity (textural properties)

Scanning electron microscopy (SEM) showed that the magnesium containing materials all had a similar morphology, whereas the AlPO-5 material had more rounded particles (Fig. 2b (C), Fig. S11 (AS)).† The magnesium content seems to cause preferred growth along one axis creating the prism shape (with flat surfaces) of MgAlPO-5(OAc) and MgAlPO-5(S). These prism shapes can still be seen in the (c-Mg)AlPO-5 material but elongation in a preferred direction is less evident. The AlPO-5 material on the other hand shows very spherical particles suggesting no additional growth in a single preferred direction. Interestingly, the surface morphology differed between MgAlPO-5(OAc) and MgAlPO-5(S); the material synthesised from struvite, in both AS (Fig. S11†) and calcined (C) forms (Fig. 2b), features thin ‘nanorod-like’ growths on the surface, which are 100's of nm in length. They can also be seen around the edges of the particles using transmission electron microscopy (TEM) (Fig. 2c for calcined MgAlPO-5(S), Fig. S12† for all other materials). Although the length is difficult to measure, the width has been measured from TEM images (Fig S13†). The average width of the growths is 33.5 (±4.8) nm. To ascertain the composition of these growths TEM-EDS images were taken. The elemental maps show that the growths contain Al, P, O and Mg (Fig. 2c, further TEM-EDS maps can be found in Fig. S14†). Owing to no other differences between MgAlPO-5(OAc) and MgAlPO-5(S) except the magnesium source, struvite must cause these growths however it remains unclear why. It could indicate the formation of another MgAlPO phase, but the growths remain too small to be identified through PXRD. Electron diffraction however could be explored to obtain unit cell parameters for these nano-sized growths. They have a different morphology when compared to struvite (Fig. S1†), so it is unlikely that the material is unreacted struvite. Further, to support this, no additional peaks, representative of struvite environments^68,69^ can be seen in the SSNMR spectra, or PXRD patterns.

Surface area measurements could reveal if using struvite as a reagent causes any differences within the pore system of the aluminophosphate. All calcined materials exhibit an isotherm which is a combination of Type IV(a) and Type I^70^ character indicating they possess both microporous and mesoporous character (Fig. S24†). Each isotherm also features an H4 hysteresis loop which is common for aggregated crystals of zeolites.^70^ The hysteresis loop of the AlPO-5 isotherm is more akin to an H3 type, possibly suggestive of a different, but still aggregated particle shape. The hysteresis could also be indicative of the presence of mesopores, or intracrystallite mesoporosity.^71^ This is supported by the observation of aggregated particles in the SEM micrographs (Fig. 2b). The BET surface areas are within those expected for aluminophosphate materials (Table S10†).^17,72,73^ MgAlPO-5(S) has the lowest BET surface area and t-plot micropore volume suggesting struvite, or related decomposition compounds,^74,75^ could be responsible for blocking pores within the material. CHN results suggest the template was completely removed (Fig. S10†), so this is unlikely to be the cause. Extra-framework magnesium could also be responsible for the decrease in surface area seen in MgAlPO-5(S).

### 3.4. Local environment

The ^27^Al SSNMR spectra of all calcined materials showed three peaks (Fig. 3, top). The largest peak corresponds to the tetrahedral aluminium environment {Al(4P)}. The peak is shifted downfield from *δ* = 35 ppm to *δ* = 38 ppm upon calcination. For Mg-containing materials this is observed, regardless of the magnesium source or content. This is contrary to the {Al(4P)} peak for AlPO-5 material which shifts upfield. The shift of this environment in the calcined material has been observed previously.^76^ The smaller peak at *δ* = −13 ppm increases in intensity when the materials are calcined. This indicates the presence of octahedrally coordinated aluminium, likely due to the coordination of water molecules upon removal of the DMCHA template.^77^ Fyfe *et al.* used two-dimensional ^27^Al → ^31^P transferred-echo double-resonance (TEDOR) SSNMR (Fig. 6, page 53, in Fyfe *et al.* (ref. 78)) to confirm that the octahedrally coordinated aluminium peak is directly linked to phosphorus within the framework. This finding suggests that six-coordinate Al is an integral part of the structure rather than an extra-framework species.^78^ An increase in intensity of this peak was again observed by Meinhold *et al.* when the volume of water mixed with AlPO-5 materials was increased.^77^ All materials featured a peak between *δ* = 0–20 ppm which can be attributed to penta-coordinate aluminium.^78,79^ This peak is smaller in the AlPO-5 material, suggesting that penta-coordinate aluminium species can arise due to the substitution of magnesium into the framework, or with increased water content.

**Fig. 3:**
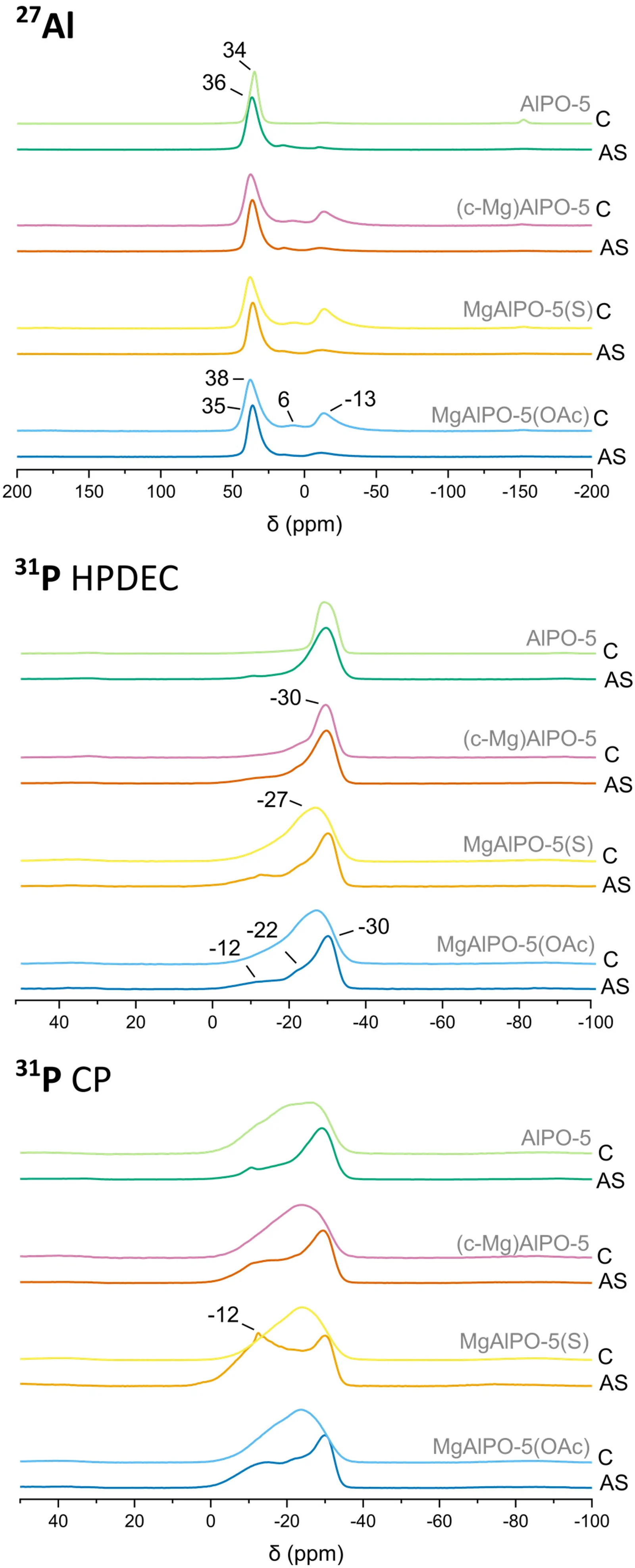
Top – ^27^Al SSNMR spectra of all four materials, both the as-synthesised (AS) and calcined (C) materials; middle – ^31^P High Power Decoupling (HPDEC) spectra of all four materials, both AS and C; bottom – ^31^P Cross Polarization (CP) spectra of all four materials, both AS and calcined. All labelled peaks are discussed in the text (section 3.4).

The ^31^P SSNMR spectra contain many overlapping peaks (Fig. 3). The ^31^P HPDEC spectra (Fig. 3, middle) of the AS materials contains a peak centred at *δ* = −29 to −30 ppm (ref. 80) with a shoulder at *δ* = −22 ppm as observed previously.^78,81^ This shoulder is more pronounced in the magnesium-containing materials than in AlPO-5. There is a third less intense peak centred at *δ* = −12 ppm. This peak becomes significantly enhanced in the CP spectra (Fig. 3, bottom) suggesting this phosphorus environment is in a region of high ^1^H density. This is most likely interactions with water or the large MDCHA template, rather than an indication of Brønsted acidity. The ^31^P HPDEC and ^31^P CP spectra of the calcined materials show similarly broad peaks, indicative of hydrated materials.^77^ While all materials show a broad peak in the ^31^P CP spectra, differences do arise in the ^31^P HPDEC spectra. Both calcined MgAlPO-5(OAc) and MgAlPO-5(S) show a broad peak centred at *δ* = −27 ppm, whereas (c-Mg)AlPO-5 and AlPO-5 show comparatively sharp peaks centred at *δ* = −30 ppm. This indicates a greater distribution of different phosphorus environments, which could occur due to Mg insertion creating a more hydrophilic framework, or framework disorder upon Mg substitution. If a greater level of hydration was solely the cause this would be expected to be reflected in the ^31^P CP spectra; the peak at *δ* = −27 ppm for MgAlPO-5(OAc) and MgAlPO(S) would be enhanced when compared to (c-Mg)AlPO-5 and AlPO-5 due to increased interactions between ^31^P and ^1^H, however, this is not the case. Others have assumed that the peak with the most negative chemical shift (*δ* = ∼−30 ppm in this case) to be representative of {P(4Al)}, with increasing downfield shifts reflecting magnesium substituted environments,^76,82,83^ as seen in the shoulder^84^ (*δ* = −22 ppm) here. As suggested by Rakoczy *et al.*, the complex spectra where P nuclei are interacting with water, template (in AS materials) and other impurities, means definitive assignment is challenging.^81^

### 3.5. Framework stability and water content

Thermogravimetric analysis (TGA) of the calcined materials shows the total mass loss of both MgAlPO-5(OAc) and MgAlPO-5(S) materials was approximately 16 wt%, a greater amount than (c-Mg)AlPO-5 which had a total mass loss of 9 wt%. The AlPO-5 has the lowest mass loss (approximately 4 wt%, Fig. S15,† top). The greater mass loss from the Mg-containing materials is ascribed to the greater affinity of water for the acidic frameworks,^85^ since the mass loss occurred consistently at a temperature of between 65–75 °C (Fig. S15,† bottom). The total mass loss of the calcined materials correlates linearly (*R*^2^ = 0.992) with Mg content (Fig. S16†). No further mass losses were recorded suggesting all four calcined materials, regardless of metal doping, are stable up to at least 800 °C. All samples had a consistent thermal history and were stored in the same manner.

The mass loss during calcination of the AS materials was also monitored using TGA (Fig. S17†). There were two mass loss events for all materials during the two temperature ramps from RT to 80 °C, and 80 °C to 120 °C. The longer temperature ramp from 120 °C to 550 °C showed that all materials lose mass gradually throughout that period of temperature increase.

To gain further insight into this gradual mass loss the DTGA was calculated and plotted (Fig. S17,† bottom), however the process is complex. All mass losses between 120 and 550 °C in the TGA are thought to be due to the complex decomposition of the MDCHA template (such as protonated or deprotonated forms). The MgAlPO-5(S) material does show an obvious additional mass loss event at approximately 175 °C (Fig. S17,† bottom). This could be suggestive of different interactions between the template and framework, or the removal of additional material remaining in the framework from the struvite, such as nitrogen (ammonium) or phosphate compounds.

### 3.6. Acid site characteristics

The DRIFTS spectra of the calcined, and subsequently dehydrated (*in situ*) samples display consistent spectral features (Fig. S18–21†), although some differences are observed. Below *ν̃* = 2500 cm^−1^ there are small differences in the bands assigned to overtone and combination vibrations of the lattice, particularly between *ν̃* = 1500–1200 cm^−1^. Other subtle differences lie between *ν̃* = 3800–3300 cm^−1^ (Fig. 4). The strong band at *ν̃* = 3673 cm^−1^, present in all four materials, is attributed to terminal P–OH hydroxyl groups.^17,86^ Two smaller bands can also be seen in all materials, albeit most clearly in AlPO-5 at *ν̃* = 3789, 3760 cm^−1^. These bands are often mentioned but seldom assigned, however, the latter has been attributed to Al-OH hydroxyl groups.^87^ Another band becomes visible in the AlPO-5 spectrum at *ν̃* = 3729 cm^−1^ when baseline corrected (Fig. 4, inset) and has also been assigned to Al–OH terminal groups previously.^17^ Interestingly, neither of the peaks at *ν̃* = 3760 cm^−1^ or *ν̃* = 3725 cm^−1^ are diminished in intensity in the presence of CD_3_CN (Fig. S23†). Lastly, a broad band extending from the P-OH hydroxyl peak at *ν̃* = 3673 cm^−1^ to *ν̃* = 3400 cm^−1^ is a result of bridging hydroxyl sites,^17,86^ which are formed upon substitution of magnesium into the framework, indicative of Brønsted acidity. The broadness of this peak is a result of the hydrogen bonding interactions of these protons.^17^ The intensity of this peak is reduced in (c-Mg)AlPO-5, and not present in AlPO-5, thus correlating with Mg content.^86^ This trend can be seen more clearly in the baseline corrected spectra (Fig. 4, inset).

**Fig. 4:**
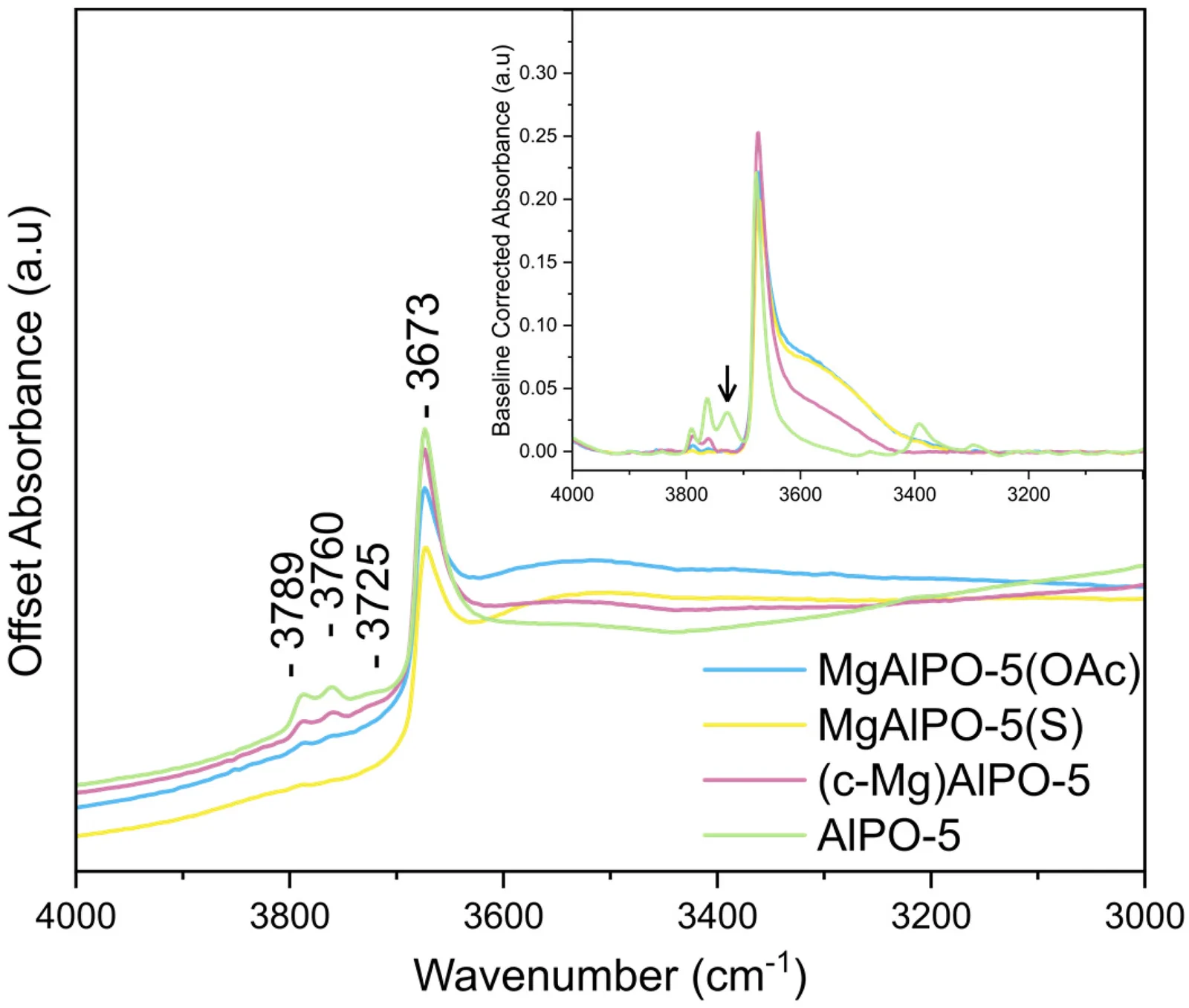
Main – DRIFTS spectra of all four (calcined) materials. Inset – baseline corrected spectrum showing the broad peak between 3600–3450 cm^−1^, representative of bridged hydroxyls more clearly. All labelled peaks are discussed in the text (section 3.6).

To further investigate the acid sites in the four calcined materials CD_3_CN was used as a probe molecule. Fig. 5 shows spectra (2500–2000 cm^−1^) for all materials; the calcined framework (black dotted line) is shown prior to CD_3_CN addition. Spectra are then recorded after 1 min of CD_3_CN exposure, before desorption spectra are recorded under dynamic vacuum at 5-, 30- and 60 min post- exposure. The C≡N stretching mode of physisorbed acetonitrile produces a peak at *ν̃* = 2265 cm^−1^ (close to free acetonitrile, Fig. 5).^88^ The magnesium containing materials (MgAlPO-5(OAc), MgAlPO-5(S), (c-Mg)AlPO-5), all show similar interactions with the probe, whereas AlPO-5 is different (Fig. 5). The sorbed CD_3_ symmetric stretch appears at *ν̃* = 2115 cm^−1^ and decreased in intensity across the desorption period.^87,88^ The sorbed CD_3_ symmetric stretch is shifted to *ν̃* = 2119 cm^−1^ in the AlPO-5 material as observed previously.^87,88^ The first of the two most prominent central peaks in all spectra associated with Mg-substituted materials is *ν̃* = 2283–2285 cm^−1^ which show CD_3_CN interacting with terminal phosphanols (also Fig. 5 inset) and bridged hydroxyl groups (suggestive of Brønsted acidity), as seen previously.^17^ This peak is shifted in the AlPO-5 to *ν̃* = 2281 cm^−1^ and decreases more rapidly in intensity during the desorption period when compared to the magnesium-substituted materials,^87^ suggesting a weaker and more reversible interaction, as noted previously by Mortén *et al.*^17^ This correlates with the lack of Brønsted acid sites in AlPO-5 since the probe interacts with these sites more strongly than terminal phosphanols.^17^ The second peak at *ν̃* = 2302–2304 cm^−1^ is the probe interacting with Mg^2+^ in the framework.^17^ The intensity of this peak is lessened in the spectra associated with (c-Mg)AlPO-5, reflecting the lower Mg content. Lastly, there is a very weak peak at *ν̃* = 2252 cm^−1^ associated with the CD_3_ anti-symmetric stretch.^87,88^ A weak peak at *ν̃* = 2325 cm^−1^ is visible in the AlPO-5 spectra suggesting an interaction of CD_3_CN with the Al^3+^ sites.^87^ Interestingly, this cannot be seen in any of the other spectra; however, since it is weak, it may be obscured by the intense peak at *ν̃* = 2302–2304 cm^−1^ seen in the MgAlPO-5 materials.

**Fig. 5:**
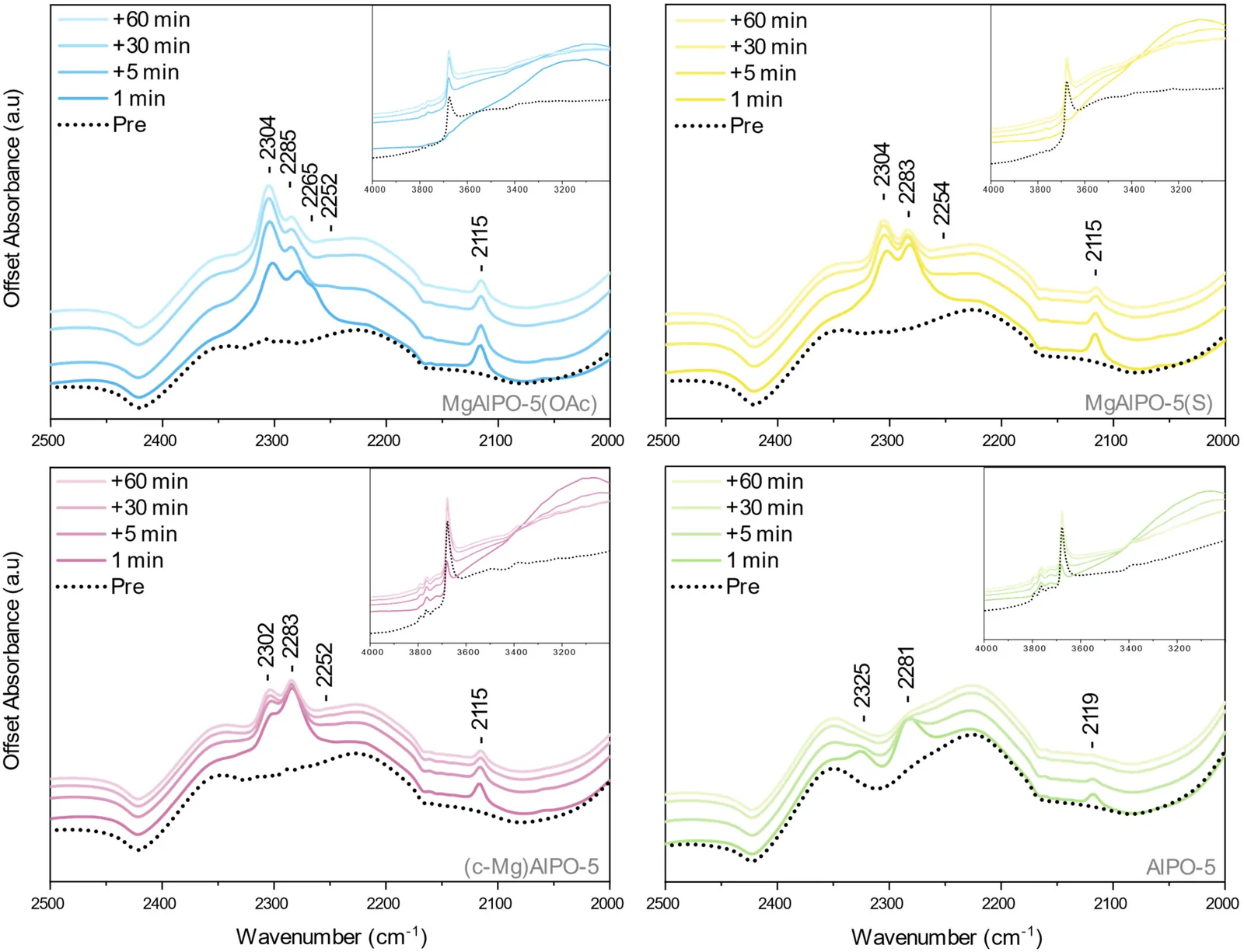
Main – DRIFTS spectra between 2500–2000 cm^−1^ using CD_3_CN to probe acid sites. The calcined framework is shown in dotted black, and the colours lighten to show the desorption process, with increasing time from acetonitrile addition into the DRIFTS cell. The cell was exposed to acetonitrile for 1 min, and a spectrum immediately recorded, before being recorded 5-, 30- and 60-minutes post addition under dynamic vacuum. Inset – region of the spectra between 4000–3000 cm^−1^ showing the P–OH peak. All labelled peaks are discussed in the text.

### 3.7. Catalysis

The dehydration of ethanol to ethylene is a well-established process that occurs with solid acid catalysts.^89–94^ Hence, this reaction was used to test the acidity of the aluminophosphate materials (MgAlPO-5(OAc), MgAlPO-5(S) and AlPO-5) synthesised here.

For all materials, ethylene was produced after the catalyst bed was held at 200 °C for 1 h (Table S11 and Fig. S25† for representative GC traces). However, the amount of ethylene produced varied between the materials. At 200 °C, MgAlPO-5(OAc) produced the most ethylene, 0.01940 mmol min^−1^ g^−1^, with MgAlPO-5(S) producing roughly half as much, 0.00937 mmol min^−1^ g^−1^. As both MgAlPO-5(OAc) and MgAlPO-5(S) contain very similar amounts of Mg (the difference in activity is significant (1.078 mmol min^−1^ Mg^−1^*vs*. 0.487 mmol min^−1^ Mg^−1^)). This suggests greater accessibility of Brønsted acid sites in MgAlPO-5(OAc). The AlPO-5 material produces significantly less ethylene, at 0.00183 mmol min^−1^ g^−1^. These results confirm that, as previously reported,^17,86,95^ MgAlPO-5 materials have a higher degree of Brønsted acidity than AlPO-5, and supported by the DRIFTS analysis in this study (section 3.6). The low production of ethylene by AlPO-5 could be due to Al–OH and P–OH groups; they have been shown to be acidic enough to effect n-butane isomerisation,^95^ and dehydrate a range of alcohols.^96^ The magnesium materials also differ despite both possessing Brønsted acidity; MgAlPO-5(OAc) shows greater activity than MgAlPO-5(S). Even considering the lower surface area of the MgAlPO-5(S) when compared to MgAlPO-5(OAc), this would not seem to account for the decrease in ethylene production. The lower rate could be indicative that the surface growths (observed by electron microscopy and shown to reduce the surface area and micropore volume of the material) are causing pore blockages and consequently diffusion limitations, resulting in a lower rate of ethylene production.

## 4. Conclusions

In this work we have successfully shown that a magnesium-substituted aluminophosphate can be synthesised from an unconventional yet sustainable source of magnesium. The MgAlPO-5 synthesised from struvite possessed the intended AFI framework structure and similar structural stability, up to at least 800 °C, the same as the conventional material synthesised from magnesium acetate. No additional disorder within the AFI framework arising from the use of the waste magnesium source is observed in the SSNMR spectra, although correlated ^27^Al–^31^P experiments would provide a more in-depth analysis. Furthermore, DRIFTS measurements showed the presence of bridging hydroxyls, indicative of Brønsted acidity, regardless of the magnesium source. These were probed further using acetonitrile demonstrating that both MgAlPO-5(OAc) and MgAlPO-5(S) contained magnesium in the framework and associated bridged hydroxyls. This is supported by the DRIFTS/CD_3_CN spectrum of AlPO-5. There is a greater degree of reversibility when acetonitrile interacts with the hydroxyl groups, which is suggestive of the presence of weakly acidic terminal phosphanols, rather than Brønsted acidic protons. However, differences arose when the particles were imaged using electron microscopy. There were small nanoparticle growths on the surface of the MgAlPO-5(S) particles. The EDS-TEM revealed that the growths contained Al, O, P and Mg but the structure remains unresolved. The BET surface area and t-plot micropore volume of MgAlPO-5(S) is also reduced, which could suggest these growths lie not only on the surface but in the porous architecture also. Despite this, MgAlPO-5(S) was capable of converting ethanol to ethylene in a flow set-up, demonstrating it does possess catalytic properties. The rate of ethylene production was lower than MgAlPO-5(OAc), possibly due to pore blockage, and hence diffusion limitations caused by these growths visible on the surface.

In conclusion, a waste source of magnesium, prevalent in industries that handle nutrient-rich waste streams, can be utilised as a chemical reactant. Mg and P are both critical raw materials, therefore it is important that they can be recovered from waste, as shown here. In the future, we hope to combine multiple waste materials to synthesise AlPOs wholly from waste. The study here demonstrates the sustainable and creative use of waste materials in the chemical sciences.

## Author contributions

CC: investigation (lead), formal analysis, visualization and writing – original draft, review and editing. JB: investigation, formal analysis, visualisation and writing – original draft, review and editing. PC: investigation, formal analysis and visualization. JSOE: formal analysis and validation, writing – review and editing. HCG: supervision and conceptualization, writing – review and editing. RT: supervision (lead), formal analysis, conceptualization and writing – original draft, review and editing.

## Conflicts of interest

There are no conflicts to declare.

## Supplementary Material

DT-054-D5DT00694E-s001

## Data Availability

The authors confirm that the data supporting the findings of this study are available within the article and the ESI,† and the raw data are available from the corresponding authors upon reasonable request.
