# Supplementary material for: A “waste” of time?: the synthesis of a magnesium-substituted aluminophosphate from an unconventional source of magnesium
Source: Dalton Trans. 2025 May 19;54(23):9250–60. doi: 10.1039/d5dt00694e (PMC13725692; doi:10.1039/d5dt00694e)
Supplement: Supplementary file 1 [file DT-054-D5DT00694E-s001.pdf]

Supplementary Information (SI) for Dalton Transactions.
This journal is © The Royal Society of Chemistry 2025

Supporting Information

for:

A "waste" of time? The synthesis of a magnesium-substituted aluminophosphate from an unconventional source of magnesium.

Catherine H. Crockett,[a] Jessica R. Bedward,[a] Phoebe K. Clayton,[a] John S. O. Evans,[a] H. Christopher Greenwell,[b] and Russell A. Taylor*[a]

[a] *Department of Chemistry, Durham University, Durham, DH1 3LE, UK*

[b] *Department of Earth Sciences, Durham University, Durham, DH1 3LE, UK*

* Corresponding Author: russell.taylor@durham.ac.uk

This file (35 pages) contains:

Text detailing the synthesis method and additional characterisation information

Supporting Figures S1 – S25

Supporting Tables S1 – S11

Reagents

All reagents were used as received without further modification and are detailed as follows: aluminium hydroxide hydrate ($Al(OH)_3 \cdot xH_2O$, Sigma Aldrich, 26 wt% H_2O as determined by thermal analysis), magnesium acetate tetrahydrate ($Mg(CH_3OO)_2 \cdot 4H_2O$, Sigma Aldrich, ACS reagent ≥ 98 %), struvite ($MgNH_4PO_4 \cdot 6H_2O$, Alfa Aesar, 98 %), D.I. H_2O (in-house supply), orthophosphoric acid (H_3PO_4, Fisher Chemical, analytical reagent grade, 85.3 % assay), N,N-dicyclohexylmethylamine ($(C_6H_{11})_2NCH_3$), Apollo Scientific, 97 %), aluminium isopropoxide ($Al(OCH_2CH_3CH_3)_3$, Sigma Aldrich, ≥ 98 %), hydrochloric acid (HCl, Fisher Chemical, 1.18 (~ 37 %)), ethanol (absolute, Fisher Scientific UK, 99.8%) and SiC (technical grade, approx. 80 grit, Fisher Scientific UK)

Full experimental details

MgAlPO-5 was prepared according to the method described by Sanchez-Sanchez et al.[1] Masses and mol of reagents can be found in the table at the end of the method. A detailed description of the synthetic procedure carried out is as follows: a beaker (Azlon, 250 mL volume) containing a stirrer bar with pivot ring (length: 35 mm, diameter: 6 mm) was placed on a stirrer hotplate. Both D.I. H_2O and H_3PO_4 were added to the beaker at a stirring speed of 600 RPM with no heat applied. A portion of $Al(OH)_3 \cdot xH_2O$ was added all at once and a lid placed on the beaker. This was stirred for 10 min at 600 RPM, with time beginning after the addition of the reagent was completed. A suspension (struvite), or aqueous solution ($Mg(CH_3OO)_2 \cdot 4H_2O$) of the magnesium source was then added dropwise to the beaker with a pipette, and the mixture stirred for a further 10 min at 600 RPM, with time beginning after the addition of the reagent was completed. Lastly, N,N-methyldicyclohexylamine (MDCHA) was added dropwise with a pipette and the mixture stirred for 90 min at 600 RPM, with time beginning after the addition of the reagent was completed. The lid was replaced on top of the beaker between the addition of each reagent. The final molar ratio of the gel is 0.74 Al: 1.50 P:0.8 MDCHA: 38.5 H_2O: 0.03 Mg (water in $Al(OH)_3 \cdot xH_2O$ and H_3PO_4 are taken into account, see table below for more details). Aluminium isopropoxide, $Al(OCH_2CH_3CH_3)_3$, was used in the above method instead of $Al(OH)_3 \cdot xH_2O$ since the latter contained small amounts of magnesium (~1.5 mol%).

The resultant opaque white gel was divided evenly between six Teflon lined autoclaves (40 mL internal volume). The mass of each was recorded, and the pH and temperature measured (see notes below) with a pH meter (ThermoScientific Orion Star A211). A cylindrical stirrer bar (length: 25 mm, diameter: 6mm) was added to each liner. The liners were sealed into the autoclaves (6 screw closure) and placed in a pre-heated oven (Carbolite Gero forced convection oven) at 180 °C on a stirrer plate (2mag MIXcontrol 20) with six positions set at 600 RPM. After 2 h the autoclaves were removed and cooled on ice for 30 min. After cooling each liner was removed from the autoclave and the resultant mixture was poured into a 50 mL centrifuge tube and topped up to 40 mL with D.I. H_2O. The tubes were centrifuged (Thermo Scientific™ Heraeus Megafuge™ 8, HIGHConic III Fixed Angle Rotor) at 6000 RPM for 5 min. The pH of the separated liquor was measured and discarded. To the solid remaining in the centrifuge tube, 40 mL of D.I. H_2O was added and shaken on a vortex mixer (Fisherbrand ZX3) to agitate the contents. The centrifuge tubes were centrifuged again for 5 min at 6000 RPM. The pH of this liquor was also measured before being discarded. The total amount of D.I. H_2O for washing totals approximately 0.5 L. The solid products were left in the centrifuge tubes (unsealed) and oven-dried at 80 °C overnight. The liners were cleaned with NaOH (2M) in the oven, with stirring, for at least 12 h between each reaction.

After PXRD and Rietveld analysis to confirm the similarity of the materials from individual autoclaves samples were combined to produce a single material prior to calcination.

A portion (approx. ~5 g) of the as-synthesised (AS) product was spread thinly onto a calcination dish (diameter of rim: 130 mm) and calcined in a static air furnace (Carbolite CWF1100). The calcination procedure is as follows: 80 °C for 1 h, 120 °C for 1 h followed by 16 h at 550 °C. The ramp rate used throughout is 2.5 °C/min, totalling approximately 21 h 30 min.

The values below present the average pH (and standard deviation) for each of the six autoclaves at three different stages: (1) pH of the gel before oven treatment (after dividing the gel), (2) pH of the supernatant after the first wash post-centrifugation, and (3) pH of the supernatant after the second wash post-centrifugation.

MgAlPO-5(OAc): 5.89 (± 0.04), 5.74 (± 0.08), 6.10 (± 0.06)
MgAlPO-5(S): 6.19 (± 0.01), 6.13 (± 0.09), 6.41 (± 0.13)
(c-Mg)AlPO-5: 6.00 (± 0.04), 6.04 (± 0.05), 6.27 (± 0.09)

AlPO-5: 6.69 (± 0.14), 6.64 (± 0.10), 6.73 (± 0.10)

The mass of each AS material isolated from a single gel mixture, post oven-drying, is given below. The total mass of each material (below) was calculated by sum isolated from each autoclave (each value is the sum of 6 small values).

MgAlPO-5(OAc): 11.7695 g
MgAlPO-5(S): 12.3644 g
(c-Mg)AlPO-5: 10.8348 g
AlPO-5: 8.4918 g

Reagent	Mass (g)	Volume (mL)	Amount (mol)
$Al(OH)_3 \cdot xH_2O$	7.7688		0.074*
H_3PO_3 (85%)	17.24 (ρ = 1.685 gcm^{-3})	10.32	0.15**
D.I H_2O	69 (54, then 15)	69 (54, then 15)	3.8524***
$Mg(CH_3OO)_2 \cdot 4H_2O$	0.6408		0.003
Struvite	0.7320		0.003
N,N-dicyclohexylmethylamine	15.57 (ρ = 0.912 gcm^{-3})	17.07	0.08
$Al(OCH_2CH_3CH_3)_3$	15.0100		0.073

* 26 wt% H_2O taken into account. 0.0260 mol H_2O added to total water mol
** 85 wt% H_2O taken into account. 0.0264 mol H_2O added to total water mol
*** Water added is 3.8 mol however 3.8524 reflects additional water from $Al(OH)_3 \cdot xH_2O$ and phosphoric acid.

Full characterisation details

Powder X-ray Diffraction

The powder patterns of all samples were recorded over a 2θ range of 2° to 50° with a step size of 0.02° on a Bruker D8 Advance diffractometer with a Cu Kα (γ = 1.54184 Å) radiation source. Samples were ground in an agate pestle and mortar, sieved through a 120 mesh sieve (0.125 mm) and adhered to a Si (9 1 1) slide in a sample holder using petroleum jelly. A knife edge was used for low angle scattering, and samples were rotated throughout data acquisition. Rietveld refinement details are provided in Tables S5-9.

Inductively-Coupled-Plasma Optical Emission Spectroscopy

The aluminium, phosphorus and magnesium content of all samples was measured on an Agilent 5800 VDV, which has a vertical touch, cyclonic spray chamber and concentric nebuliser, by Emily Unsworth, Department of Chemistry. Sample preparation was as follows: approximately 0.01 g of sample was dissolved in hydrochloric acid (1 mL, 37%) and heated for 5-10 min in a water bath at 80 °C. D.I. H_2O (9 mL) was then added.

Carbon-Hydrogen-Nitrogen Analysis

Analysis was conducted on an Exeter Analytical CE-440 elemental analyser which was calibrated with cystine. Analysis was conducted by Emily Unsworth, Department of Chemistry.

Scanning Electron Microscopy

To conduct analysis pin stubs were prepared by adhering a carbon disc to the surface, followed by a piece of clear polycarbonate. Samples were dusted on then sputter collated in gold (35-40 nm). Analysis was performed on a Zeiss Sigma 300 VP, with an accelerating voltage of between 5 and 8 kV.

Transmission Electron Microscopy

The samples were suspended and ground with isopropanol alcohol in an agate pestle and mortar before being pipetted onto a holey carbon film with Cu mesh (300). Analysis was performed on a JEOL 2100F TEM. Elemental mapping was performed using an Oxford XMax 65.

Thermogravimetric Analysis

Thermal analysis of the calcination procedure was performed under air, with a ramp rate at 2.5 °C/min throughout. The sample was heated to 80 °C, held for 1 h, heated to 120 °C, held for a further 1 h, before the final ramp to 550 °C for 16 h.

The TGA profile of calcined samples were measured under air, using a 10 °C/min ramp rate, from 30 °C to 800 °C.

Analysis was conducted on a Perkin Elmer TGA 8000 by William Carsell, Department of Chemistry.

N_2 Adsorption/Desorption Isotherms

Analysis was performed on a Micromeritics ASAP 2020. Prior to analysis all calcined samples were degassed on the instrument using the following procedure: ramp to 350 °C (10 °C/min), hold for 10 h. The isotherms were collected at -196 °C. From the isotherms the BET equation was applied in a p/p_0 range of 0.01-0.1 for all samples to determine the specific surface area. The t-plot method was used to calculate the micropore volume and external surface areas.

Diffuse Reflectance Infrared Fourier Transform Spectroscopy

Analysis was performed on a Bruker Invenio-R FT-IR equipped with an MCT detector, Harrick Praying Mantis™ high temperature reaction chamber and Harrick ATK controlled heating element. The spectrometer is fed with a constant flow of dry N_2 (0.6 L/h) to ensure no moisture build-up on the sample or detectors. To provide cooling and extend the lifetime of the chamber seal, a Huber MiniChill600 circulates water at room temperature around the cell to act as a coolant. Vacuum to the cell is initially provided by a Pfeiffer Vacuum Hi-Scroll 600 (up to 1×10^{-2} hPa), before being switched to a Pfieffer Vacuum HiCube 80 eco turbopump to reduce pressures further (~1×10^{-3} hPa). Pressure of the turbopump is measured using a Pfeiffer Vacuum PKR360 gauge, and pressure of the cell is measured using a Pfieffer Vacuum PKR360C gauge. For CD_3CN dosing two additional gauges were used: a Pfieffer Vacuum CMR361 and a Pfieffer Vacuum CMR36.

An 80-mesh stainless steel gauze (made in-house) was placed into the sample cup before the ground material was loaded on top. A thermocouple (0.5 mm diameter, K-type) has been inserted through the wall of the sample couple into the centre of the sample bed. An El-USB-TC-LCD thermocouple data logger is attached during measurements to monitor the temperature of the sample bed. Background spectra were collected using dehydrated KBr. Prior to dehydration in the Harrick Praying Mantis cell, the KBr remained in a vacuum oven (ThermoScientific Heraeus Vacuum oven) at 80-100 °C. Prior to the temperature ramp, both KBr and samples were subjected to high vacuum overnight.

Both the background and sample were subjected to the same temperature ramp from room temperature to 450 °C, at 5 °C/min. Spectra were collected approximately every 8 min. The CD_3CN experiments were performed at temperatures of between 25 °C and 30 °C. Spectra were collected as follows: 1) immediately after exposing the sample to CD_3CN for 1 min, 2) after 5 min of exposure to a dynamic vacuum reaching ~10^{-1} hPa, 3) after 30 min of exposure to higher dynamic vacuum reaching ~10^{-2} – 10^{-3} hPa, 4) then lastly after 60 min (resulting in a total 4 spectra post CD_3CN addition). Temperature discrepancies between the cartridge heater and sample cup are known,[2] so an external thermocouple, going through the sample cup into the sample bed is also used to monitor the temperature.

The spectrum processing was performed in the Bruker OPUS software and involved ratioing of sample spectrum against the KBr background spectrum to remove instrument and baseline effects. Each individual sample spectrum had a corresponding KBr background spectrum recorded at the same temperature. The spectra were then converted from reflectance to absorbance. Where stated, baseline correction was performed in the Bruker OPUS software using the "Concave Rubberband Correction". The process was performed over 10 iterations with 64 baseline points across the entire spectrum.

Solid-state Nuclear Magnetic Resonance Spectroscopy

Data acquisition and processing was conducted by Dr Samuel Page, Department of Chemistry. The samples were packed into rotors under ambient conditions.

Aluminium-27 magic-angle spinning measurements were recorded at 104.3 MHz using a Bruker Avance III HD spectrometer and a 3.2 mm (rotor o.d.) magic-angle spinning probe at a sample spin-rate of 20 kHz. All direct excitation ^{27}Al spectra were acquired with a 1 µs 30 degree solid pulse which was determined from a 9 µs solution pulse determined on 1M $Al(NO_3)_3$. The spectra were acquired with a

recycle delay of 1 s determined on the sample. Aluminium spectral referencing is relative to 1M $Al(NO_3)_3$ carried out by setting the signal to 0.0 ppm.
Phosporus-31 spectra were recorded at 161.99 MHz using a Bruker Avance III HD spectrometer and a 4.0 mm (rotor o.d.) magic-angle spinning probe at a sample spin-rate of 10 kHz. The spectra were obtained using both cross polarisation (CP) and direct excitation with high powered decoupling (HPDEC). For the CP spectra both the recycle delay and contact time were optimised on each sample and found to be between 1-2 s and 1 ms respectively for all samples. The HPDEC data was acquired with an optimised 100 s recycle delay. Spectral referencing was with respect to an external sample of Phosphoric acid (85% H_3PO_4) by setting the signal to 0.0 ppm.

Catalytic testing

A HEL FlowCat flow reactor with online GC-MS-BID was used for catalytic testing and analysis. The reactor effluent flows *via* 1/16-inch trace heated transfer lines (maintained at 170 °C) to a Shimadzu GC-2010 Plus gas chromatograph. Vici Valco GC injector valves were used for reactor effluent injection into the GC. The effluent first passed through two 6 port Vici valves held at 180 °C, and two samples were automatically removed from the effluent *via* a 0.05 mL sample loop on each Vici valve. Two separate split valves were then used to inject the respective samples into the GC, with the injection port for Sample 1 being maintained at 220 °C, and Sample 2 at 200 °C. Sample 1 was analysed using a Shimadzu GC-MSQP2010 SE mass spectrometer, with ion detection in an m/z range of 10-200, a scan speed of 666 ms and a sampling rate of 40 ms. Sample 1 was injected with a 125:1 He split ratio and chromatography performed over a Thames Restek RTX-VMS fused silica capillary column (Length 30 m, I.D. 0.25 mm, film thickness 0.25 µm). Sample 2 was analysed using a Shimadzu BID-2010 Plus detector with He plasma maintained at 300 °C and a sampling rate of 40 ms. Sample 2 was injected with a 5:1 He split ratio and chromatography performed over a Restek ShinCarbon ST packed column with a high surface area (~1,500 $m^2\ g^{-1}$) carbon molecular sieve stationary phase (length 2 m, I.D. 1.00 mm, O.D. 1/16", packed 100/120 mesh). Both columns were mounted in the same oven and hence underwent the same heating profile throughout the chromatography stage. The same GC programme was used for all samples. Samples were injected into the column at 80 °C, where temperature was held for 7.0 min. Temperature was subsequently ramped to 250 °C at a ramp rate of 30 °C min^{-1}, where the temperature was held for 18 min, giving a total run time of approximately 30 min.

Table S 1 A compilation of references detailing the magnesium source used, the framework synthesised and the year the reference was published. Two search terms were used on Web of Science: ("aluminophosphate" AND "magnesium") and ("mapo" AND "framework"). This yielded a total of 108 and 70 results respectively. They were manually inspected, and irrelevant publications, and repeated publications from the same authors where the same framework has been synthesised were removed. (MgOAc = magnesium acetate)

Mg Source	Framework Type	Year
$MgOAc{\cdot}4H_2O$	5 (AFI)	1995[3], 1996[4] , 2004[5], 2009[6] , 2024[7]
	11 (AEL)	1995[8] , 2002[9], 2007[10], 2020[11], 2022[12]
	17 (ERI)	2007[13]
	36 (ATS)	1999[14], 2008[15], 2013[16] , 2022[17]
	39 (ATN)	1995[18], 1998[19]
	43	1995[18], 2013[20]
	44	2010[21]
	53 (AEN)	2013[20]
	56 (AFX)	2007[13]
	STA-1 (SAO)	1997[22]
	STA-2 (SAT)	1997[23]
	DAF-1 (DFO)	1993[24]
	LEV	2012[25]
	Mesoporous, tubular arrays	2001[26]
	JU92-300	2013[27]
	JU102	2014[28]
	CJ2	2013[20]
	5/11 mixed phase	2016[29]
	5/36 mixed phase	2016[29]
MgOAc*	5	2008 [30]
	11	2009 [31], 2021 [32]
	20	1997 [33]
	36	2005 [34]
	39	1994 [35], 1997 [36], 2000 [37]
MgO	5/34 (CHA) mixed phase	1995 [38]
	11	2005 [39]
	36	1993 [40]
	50	1995 [41]
$MgSO_4{\cdot}7H_2O$	5	1993 [42], 1996 [4]
	11	2009 [43], 2014 [44]
	43	1994 [45]
$MgSO_4$	5	1994[46]
$MgHPO_4{\cdot}3H_2O$	Rho	1998 [47]
	56	2001 [48]
$Mg_3(PO_4)_2{\cdot}8H_2O$	UiO-20	2000 [49]
$Mg_3(PO_4)_2{\cdot}8H_2O$	UiO-28	2001 [50]
$MgCl_2{\cdot}6H_2O$	NJU	2006 [51]
	ITQ-51	2023 [52]
$MgCl_2$ anhydrous	5	2022 [53]
$Mg(NO_3)_2$	36	2008 [54]
$Mg(NO_3)_2{\cdot}6H_2O$	31	2017 [55]
	11	2022 [12]

** where only MgOAc was mentioned it is assumed to be tetrahydrate as it is the primary available solid, but these have been kept separate from references where the tetrahydrate salt was explicitly mentioned.*

Table S 2 A compilation of references detailing the specific AlPO catalyst, catalytic reaction and publication year. The results were selected from the search on Web of Science: ("aluminophosphate" AND "cataly*"). The last entry into the table demonstrates the use of AlPOs as water sorbents.

AlPO Catalyst	Catalytic Reaction	Summary
MgAlPO-5	Oxidative dehydration of ethane[46]	The activity and selectivity of the catalyst towards ethene decreased in the following

		order: MgVAlPO-5 > VAlPO-5 > MgAlPO-5 > AlPO-5. Summarised the high activity of MgVAlPO-5 was due to the proximity of acid (Mg^{2+}) and redox (V^{5+}) sites within the framework.
MgAlPO-36	Conversion of *n*-hexane, isooctane and toluene[56]	Primarily a conversion study to monitor the effects of magnesium content on the properties, and therefore catalytic activity. A greater magnesium content in the framework results in increased conversion, and selectivity towards aromatic products in reactions with n-hexane and isooctane.
MgAlPO-5	Isobutane conversion[57]	A study of acid site strength using isobutane conversion. Mg-DAF-1 and MgAlPO-5 yielded butenes with high selectivity, a reflection on their mild Brønsted and Lewis acidity. They both showed weaker acidic character than MgAlPO-36.
MgAlPO-5	*n*-butane isomerisation to isobutane[7]	A series of MgAlPO-5 with different mol% of Mg in the gel were synthesised (1, 3 and 6%). The variation in the mol% of Mg doping allows for control over the number of acid sites within the framework, and therefore correlations to catalytic performance can be drawn. The order of conversion is as follows: 3mol% > 6mol% > 1mol%. An investigation into mechanistic pathways to the product was also undertaken. Large pores of MgAlPO-5 promote a particular pathway to isobutane formation.
MgAlPO-5	Isopropylation of benzene and biphenyl[58]	Alkaline earth metals (Mg, Ca, Sr and Ba) were isomorphously substituted into the AlPO-5 framework. The acidic properties of the catalysts were tested using the isopropylation reaction. All catalysts were active in both reactions, and activity decreased in the following order: Mg >> Ca > Sr > BaAlPO-5.
MgAlPO-5	Ethylation of benzene with ethanol[59]	Ethylbenzene is utilised in the industrial production of styrene, so the production of this compound was investigated using heterogenous catalysts. The maximum conversion achieved was 47% with MnAlPO-5, with a 14% yield of ethylbenzene. The activity of the other catalysts is as follows: ZnAlPO-5 > MgAlPO-5 > AlPO-5. The grater activity of the manganese substituted variant of AlPO-5 was associated with unpaired electrons in the d-subshell of the manganese dopant.
MgAlPO-5	Methanol conversion[60]	The substitution of various metals into the AlPO-5 framework created both Bronsted and Lewis acidic sites of varying strength. The undoped AlPO-5 displayed some mild catalytic activity and was able to catalyse the dehydration of methanol to dimethylether. No further C-C bond formation occurs. Similar results were observed with Cr and Zr doped variants. Of the catalysts where aluminium was

		substituted for a divalent cation, NiAlPO-5 shows the best activity for the formation of low olefins.
MgAlPO-36	Methanol to hydrocarbons (MTH)[17]	Methanol conversion was used to test the solid acid catalysts synthesised, with metal doping into AlPO-36 with Zn, Co and Mg. The overall activity for methanol conversion decreases in the following order: Zn (99.8%) > Mg (62.4%) > Co (40.3%). ZnAlPO-36 shows greater selectivity towards the aromatic fraction, whereas both Co and MgAlPO-36 show greater selectivity towards light olefins. This was attributed to the low acidity of MG and Co variants when compared to Zn.
MgAlPO-18	Methanol to olefins (MTO) via CO_2 hydrogenation[61]	A study using a ZnO:ZrO_2 bed followed by an MAlPO catalyst, where M is either Mg or Si which mediates methanol to hydrocarbon conversion. Both catalysts promoted different mechanisms and were stabilised differently when feed conditions were changed. MgAlPO-18 contained CHA (AlPO-34) impurities within the AEI structure.
MgAlPO-5	Water adsorption[62]	Substitution of both Mg and Fe into the AlPO-5 framework, in differing amounts was undertaken to influence hydrophobic/hydrophilic behaviour. FeAlPO-5 showed greater water uptake than MgAlPO-5.

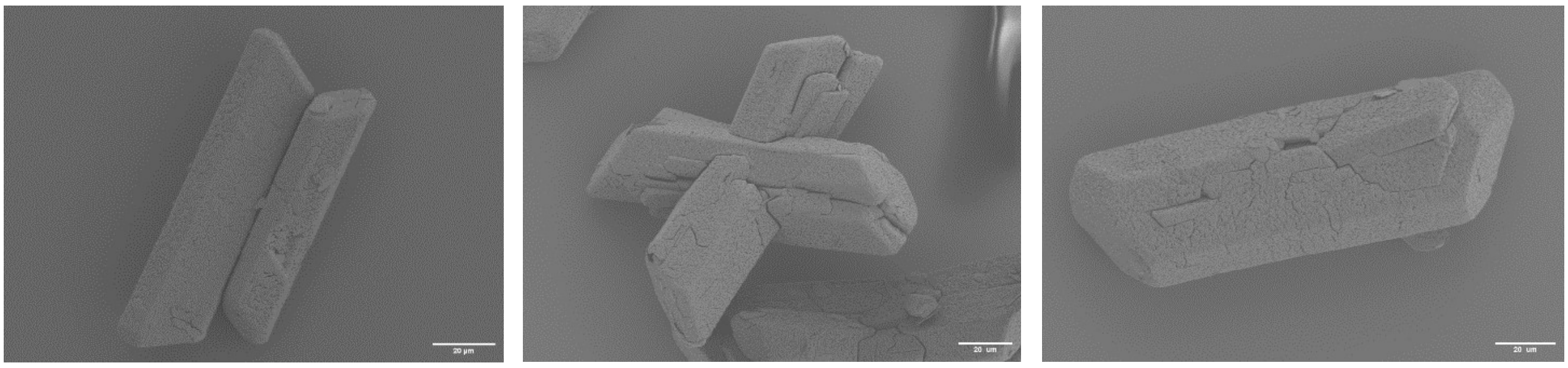

Figure S 1 SEM micrographs of synthetic struvite, showing the orthorhombic coffin-like crystals. The central micrograph shows an intergrowth of two crystals.

Table S 3 The sample names given with aluminium source and magnesium source, where applicable.

Sample Name	Aluminium Source	Magnesium Source
**MgAlPO-5(OAc)**	$Al(OH)_3{\cdot}xH_2O$	$Mg(CH_3COO)_2{\cdot}4H_2O$
**MgAlPO-5(S)**	$Al(OH)_3{\cdot}xH_2O$	$MgNH_4PO_4{\cdot}6H_2O$
**(c-Mg)AlPO-5**	$Al(OH)_3{\cdot}xH_2O$	Not added
**AlPO-5**	$Al(O\text{-}i\text{-}Pr)_3$	Not added

Table S 4 Table (accompanying Fig. S2 of PXRD patterns) demonstrating changes made to the synthesis protocol to achieve a pure AFI material. S = stirring, G = gel, T = time and Te = template. Changes to the gel are detailed in the "Actions" column, and phases formed are also shown, the primary contaminant of which is chabazite (CHA). The modifications required to avoid CHA contamination were significantly greater for struvite as the magnesium source than magnesium acetate.

Stirring

**Struvite**		
**Graph Reference**	**Action**	**Phases**
S1	Unstirred	AFI, CHA
S2	Stirred @ 600 RPM	AFI, CHA
S3	Stirred @ 1200 RPM	AFI, CHA
S4	Stirred @ 1200 RPM<br>Cross shaped	AFI, CHA

		**Magnesium acetate**
**Graph Reference**	**Action**	**Phases**
S1a	Unstirred	AFI, CHA
S2a	Stirred @ 600 RPM	AFI

Stirring

		stirrer bars instead of cylindrical				
Gel	G1	Struvite solubilised using a portion of the H_3PO_4 to be added	AFI, CHA			
Gel	G2*	As above, and temperature increase to 200 °C	AFI, CHA			
Gel	G3	Increased triethylamine amount by 50%	AFI, CHA			
Time	T1	Increased synthesis time: 3h (+1 h)	AFI, CHA			
Time	T2	Increased synthesis time : 17.5 h (+ 15.5 h)	AFI, CHA	T2a	Increased synthesis time : 17.5 h (+ 15.5 h)	AFI
Template	Te1	New template	AFI			
Template	Te2**	New template, and stirred at 1200 RPM	AFI			

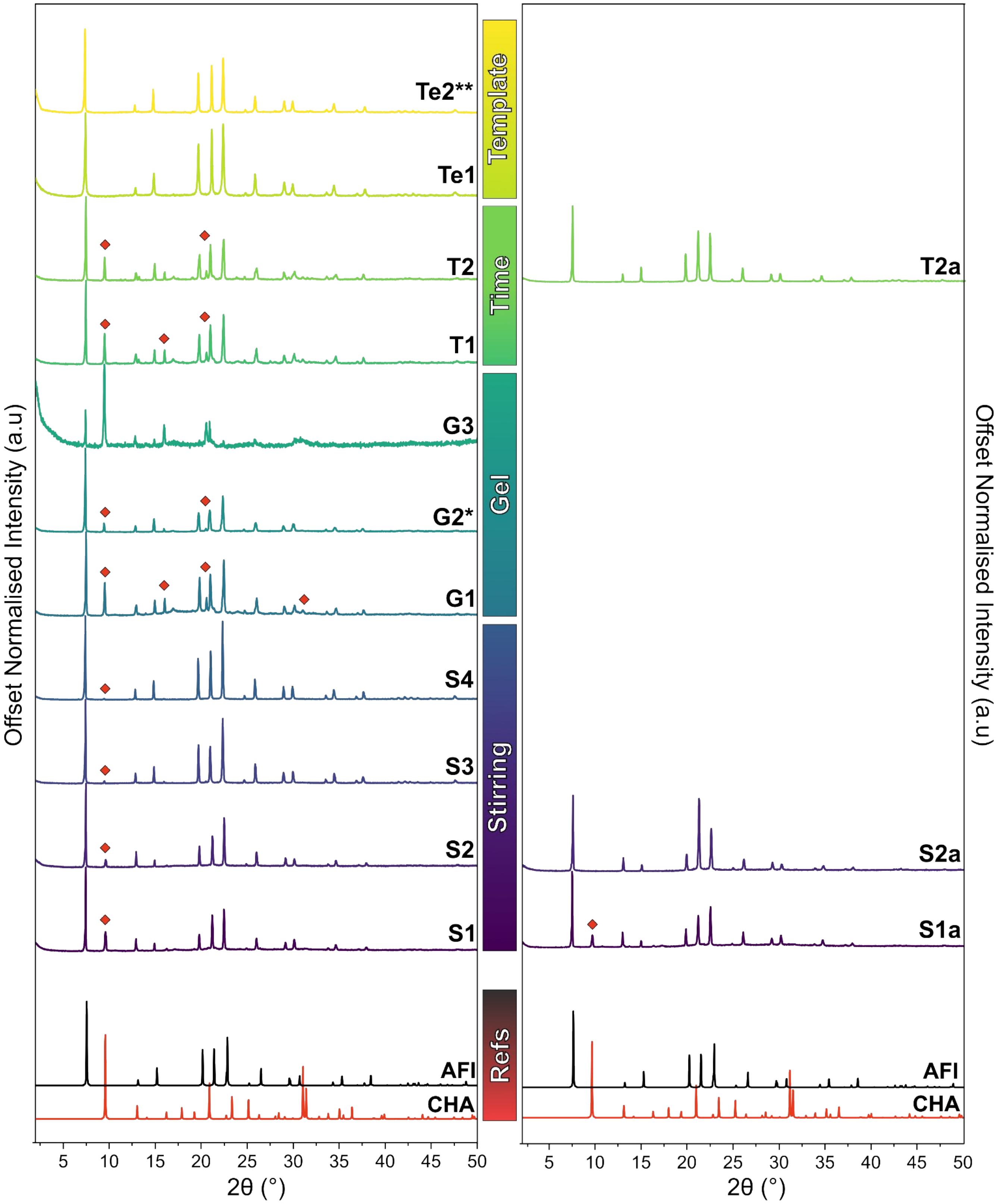

Figure S 2 Stacked PXRD patterns from each reaction listed in Table S4. The reference patterns for both AFI[67] and CHA[68] are shown below and were from the International Zeolite Associate – Structure Commission website (IZA-SC), original references as above. CHA contaminant peaks are denoted with a red diamond. The patterns are grouped dependent on the synthesis variable that was investigated; (from the bottom) stirring and stirring speed, changes to the gel or reactants, synthesis time in the oven, and lastly the template, which resulted in the synthesis of pure AFI when using struvite as the magnesium source. The specific changes can be seen in Table S4.

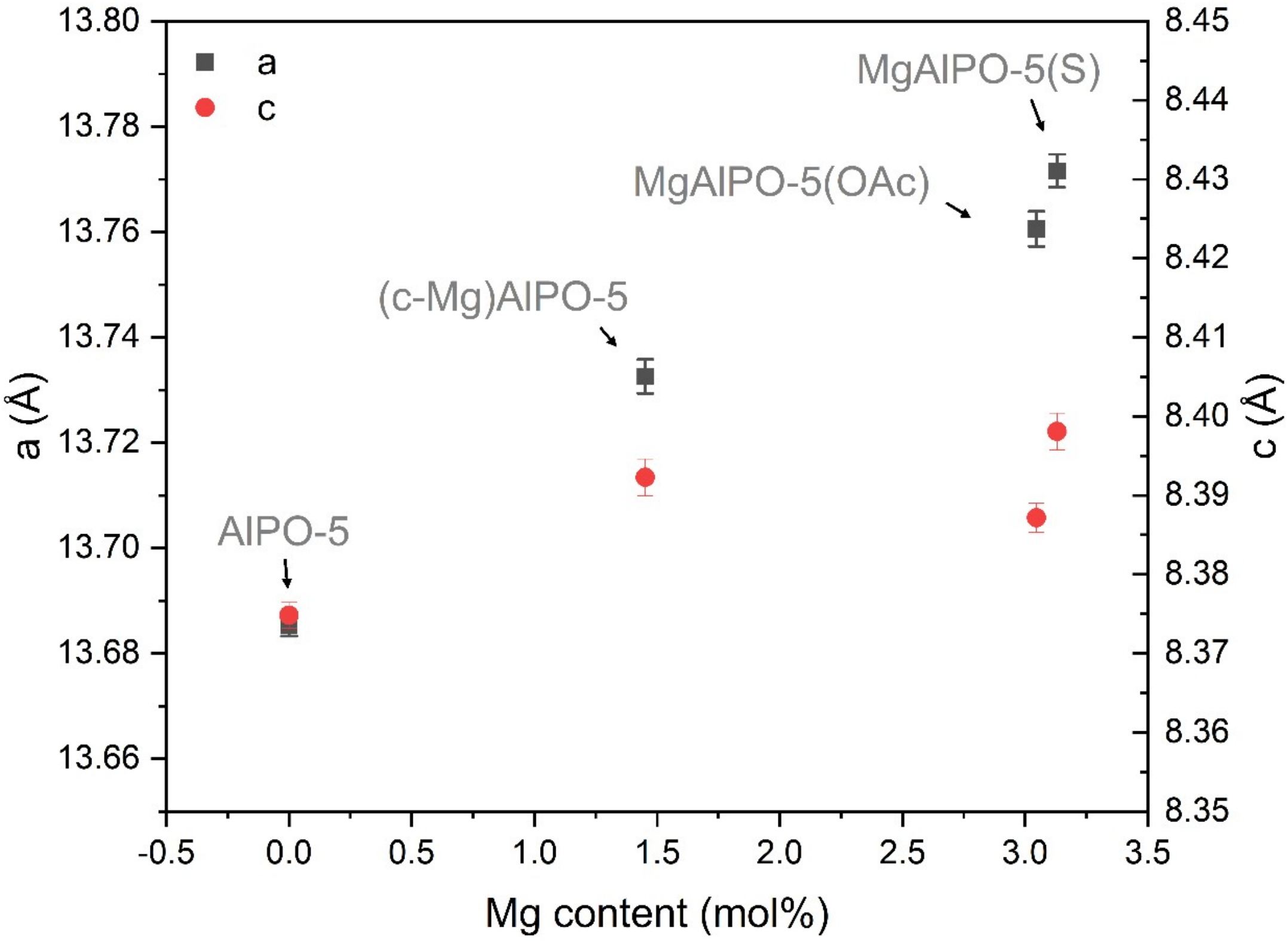

Figure S 3 Samples denoted by Mg content (mol%), from ICP-OES elemental analysis, against their *a* and *c* lattice parameters, which have been calculated from Rietveld refinement of PXRD patterns. Error bars shown were calculated using standard deviation. The correlation of the *a* lattice parameter shows a greater linear relationship to Mg content (R^2 = 0.98615) compared to the *c* lattice parameter (R^2 = 0.66532).

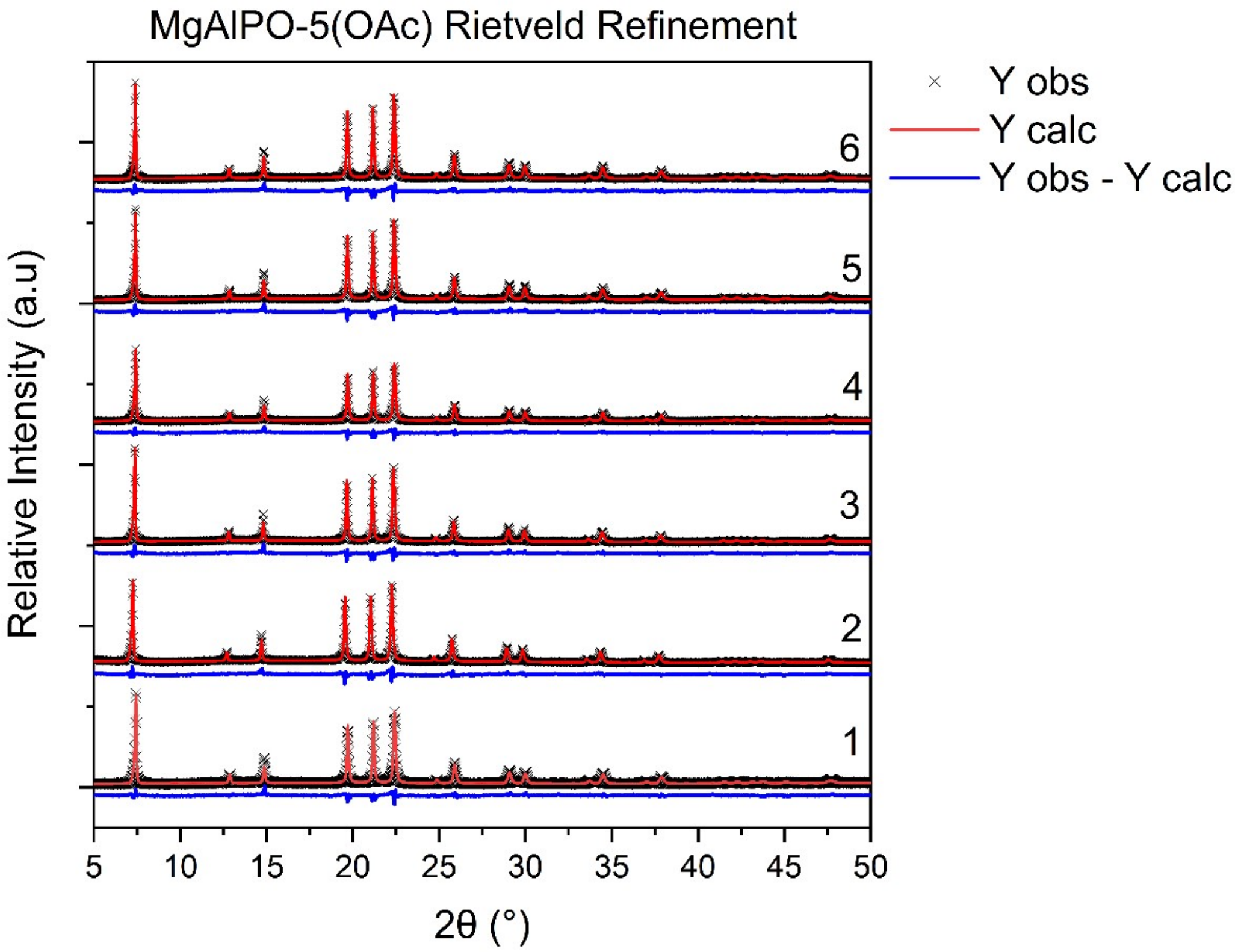

Figure S 4 Plots showing the Rietveld fitting of PXRD data from each autoclave of MgAlPO-5(OAc). Each product comprises of a single gel which was split between six autoclaves. Refinement was conducted on data before combining all AS product for calcination. Black crosses show experimental points, the red line the calculated curve and the blue line the difference between observed and calculated profiles. The model describes one P site, one Al site and four O sites for the framework, along with 5 Cl sites within the porous architecture to account for peak intensities of the hydrated sample. Anti-bumping penalties were added to the refinement to prevent the Cl atoms from getting artificially close to the framework atoms. Further refinement parameters are discussed in Table S8.

Table S 5 Cell parameters, *a* and *c*, and the weighted profile factor (R_{wp}), the profile factor (R_p), the goodness of fit (GoF) and the expected R-factor (R_{exp}) for MgAlPO-5(OAc). The standard deviation (SD) values are calculated to quantify the variation in the *a* and *c* parameters across the 6 autoclaves, rather than the fluctuations of the cell parameters during the Rietveld refinement iterations. The values correspond to samples taken from each individual autoclave, into which the batch of synthesis gel was divided.

**Autoclave No.**	***a* (Å)**	**SD**	***c* (Å)**	**SD**	**R_{wp} (%)**	**R_p**	**GoF**	**R_{exp}**
**1**	13.76326	0.00333	8.38889	0.00187	10.18423	7.923916	1.938448	5.25803
**2**	13.75411		8.38389		10.22941	8.231999	2.064344	4.955284
**3**	13.76074		8.38769		10.65117	8.356217	2.008718	5.30247
**4**	13.76179		8.38736		9.88324	7.801052	1.790775	5.518974
**5**	13.76283		8.38885		10.00772	7.814460	1.969664	5.080927
**6**	13.76075		8.38634		9.95741	7.785093	2.00556	4.964902
**AVG**	13.76058		8.387167		10.1522	7.985456	1.962918	5.179393

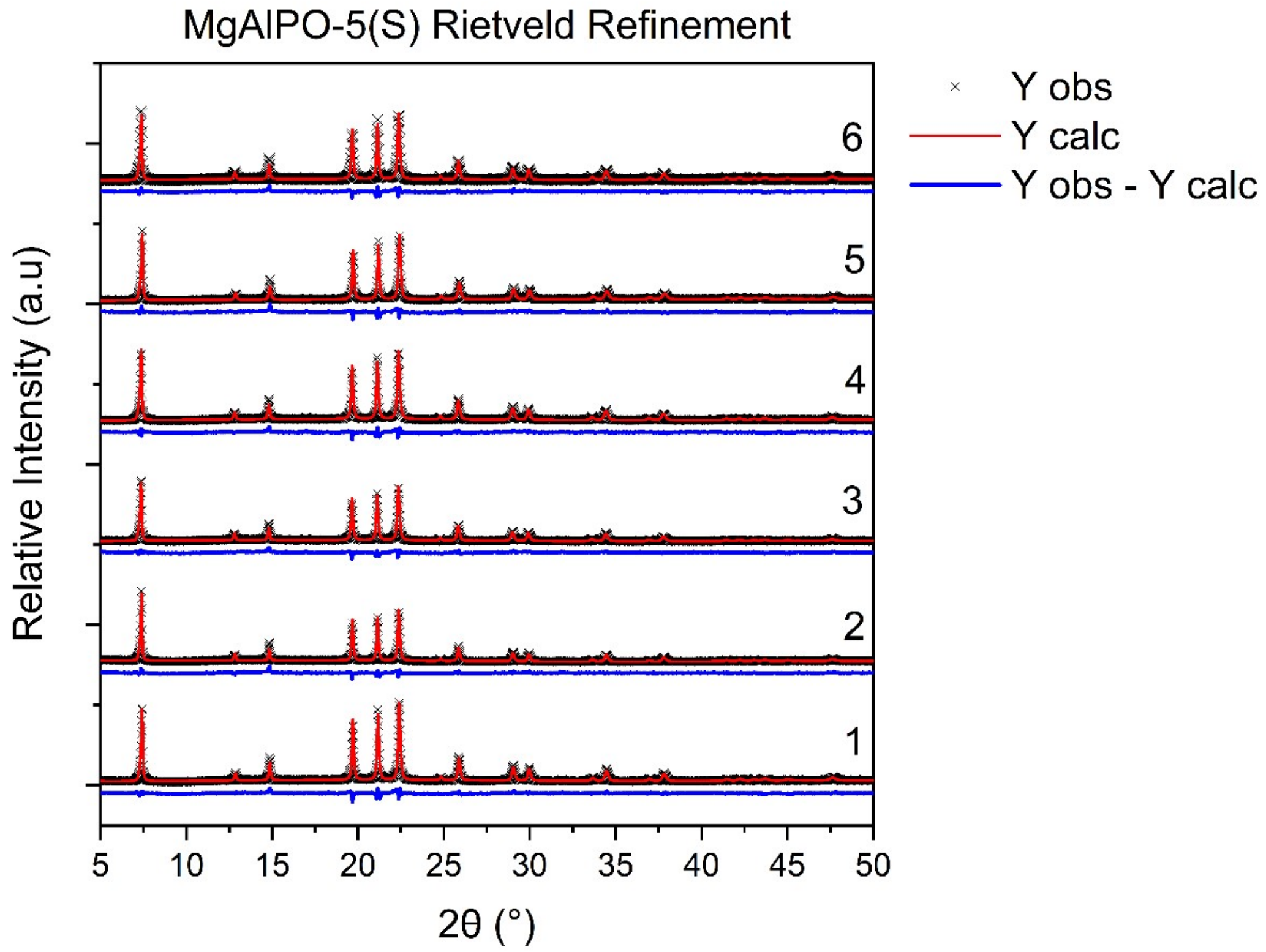

Figure S 5 Plots showing the Rietveld fitting of PXRD data from each autoclave of MgAlPO-5(S). Each product comprises of a single gel which was split between six autoclaves. Refinement was conducted on data before combining all AS product for calcination. Black crosses show experimental points, the red line the calculated curve and the blue line the difference between observed and calculated profiles. Refer to caption of Fig. S4 for model description.

Table S 6 Cell parameters, *a* and *c*, and the weighted profile factor (R_{wp}), the profile factor (R_p), the goodness of fit (GoF) and the expected R-factor (R_{exp}) for MgAlPO-5(S). The standard deviation (SD) values are calculated to quantify the variation in the *a* and *c* parameters across the 6 autoclaves, rather than the fluctuations of the cell parameters during the Rietveld refinement iterations The values correspond to samples taken from each individual autoclave, into which the batch of synthesis gel was divided.

**Autoclave No.**	***a* (Å)**	**SD**	***c* (Å)**	**SD**	**R_{wp} (%)**	**R_p**	**GoF**	**R_{exp}**
**1**	13.7776	0.00312	8.40235	0.00233	9.10221	7.144122	1.841857	4.941863
**2**	13.77096		8.39833		10.18593	7.86966	1.825752	5.579033
**3**	13.77167		8.39846		9.67642	7.522361	1.751001	5.526291
**4**	13.77109		8.39706		9.16161	7.182924	1.839007	4.981827
**5**	13.76949		8.39581		9.61001	7.384943	1.897625	5.064231
**6**	13.76888		8.39646		9.47687	7.37279	1.807279	5.243720
**AVG**	13.77162		8.398077		9.535506	7.4128	1.827087	5.222815

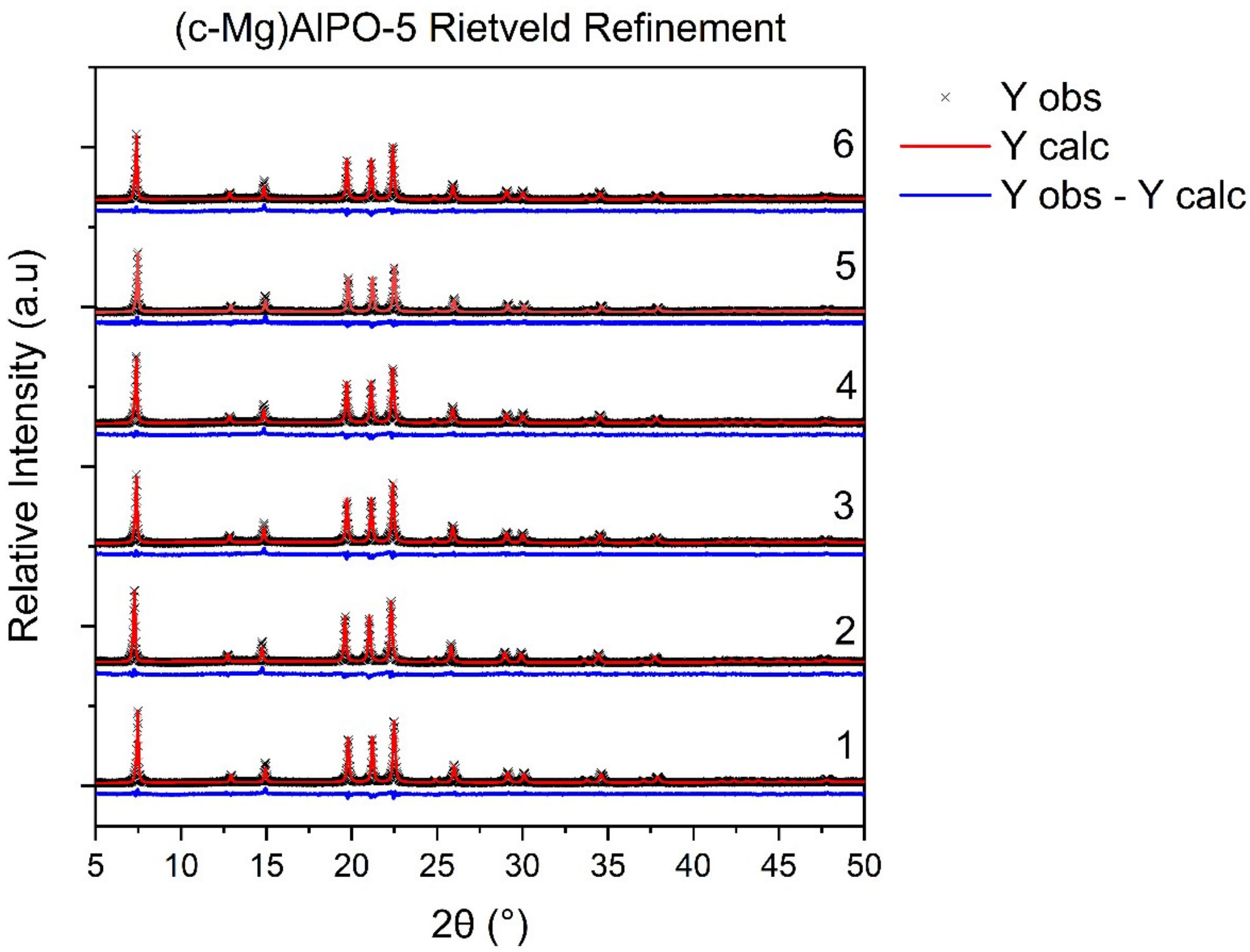

Figure S 6 Plots showing the Rietveld fitting of PXRD data from each autoclave of (c-Mg)AlPO-5. Each product comprises of a single gel which was split between six autoclaves. Refinement was conducted on data before combining all AS product for calcination. Black crosses show experimental points, the red line the calculated curve and the blue line the difference between observed and calculated profiles. Refer to caption of Fig. S4 for model description.

Table S 7 Cell parameters, *a* and *c*, and the weighted profile factor (R_{wp}), the profile factor (R_p), the goodness of fit (GoF) and the expected R-factor (R_{exp}) for (c-Mg)AlPO-5. The standard deviation (SD) values are calculated to quantify the variation in the *a* and *c* parameters across the 6 autoclaves, rather than the fluctuations of the cell parameters during the Rietveld refinement iterations The values correspond to samples taken from each individual autoclave, into which the batch of synthesis gel was divided.

**Autoclave No.**	***a* (Å)**	**SD**	***c* (Å)**	**SD**	**R_{wp} (%)**	**R_p**	**GoF**	**R_{exp}**
**1**	13.73544	0.00321	8.39609	0.00234	8.70766	6.778554	1.581881	5.504622
**2**	13.72711		8.38995		9.05800	7.125515	1.733053	5.226615
**3**	13.73459		8.39165		9.13929	7.119065	1.67721	5.449101
**4**	13.73466		8.39204		9.16598	7.193865	1.672969	5.478867
**5**	13.7304		8.39015		9.62536	7.509003	1.603848	6.001417
**6**	13.73326		8.39383		9.11662	7.151201	1.630545	5.591148
**AVG**	13.73258		8.392283		9.135483	7.1462	1.649918	5.541962

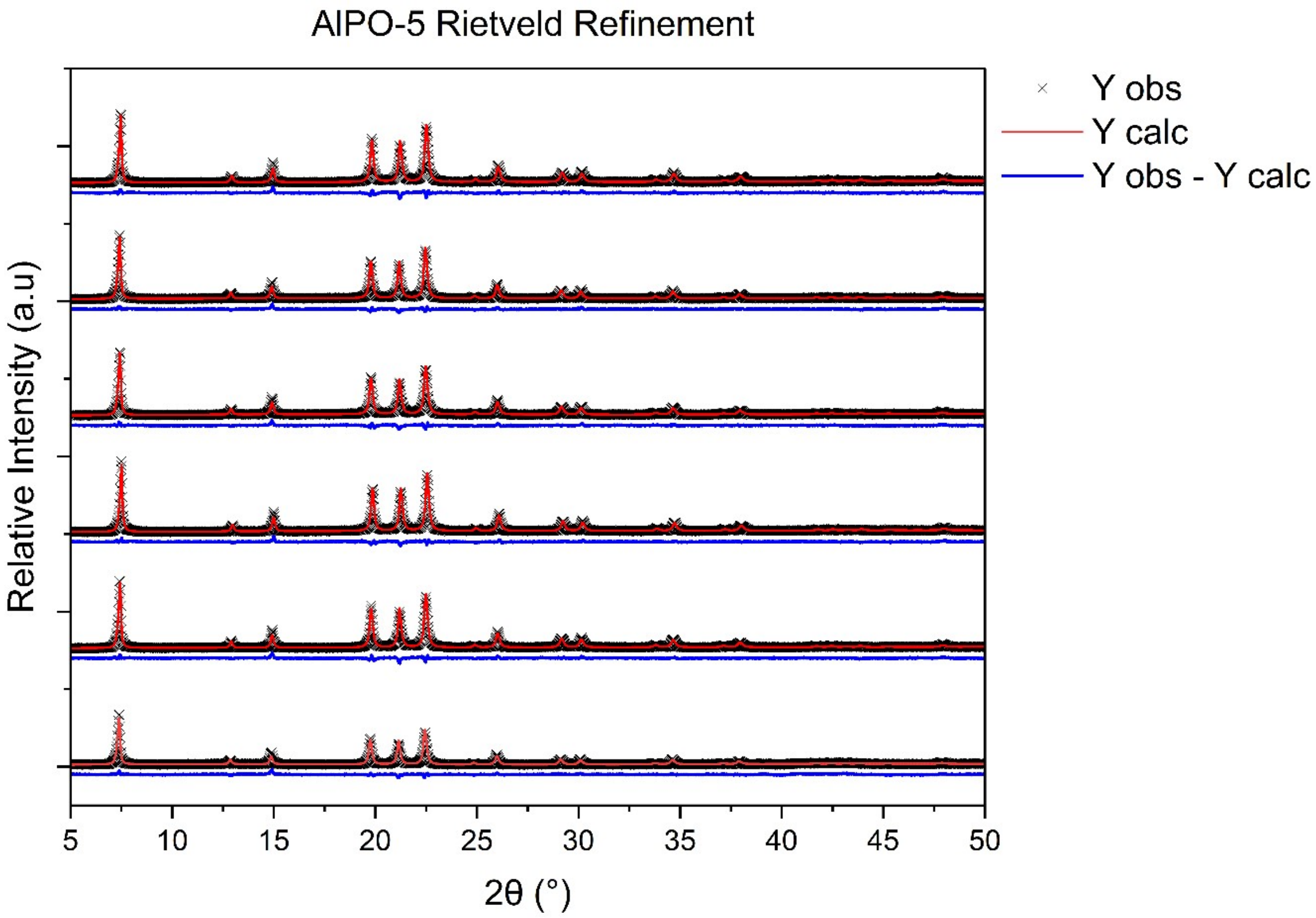

Figure S 7 Plots showing the Rietveld fitting of PXRD data from each autoclave of AlPO-5. Each product comprises of a single gel which was split between six autoclaves. Refinement was conducted on data before combining all AS product for calcination. Black crosses show experimental points, the red line the calculated curve and the blue line the difference between observed and calculated profiles. Refer to caption of Fig. S4 for model description.

Table S 8 Cell parameters, *a* and *c*, and the weighted profile factor (R_{wp}), the profile factor (R_p), the goodness of fit (GoF) and the expected R-factor (R_{exp}) for AlPO-5. The standard deviation (SD) values are calculated to quantify the variation in the *a* and *c* parameters across the 6 autoclaves, rather than the fluctuations of the cell parameters during the Rietveld refinement iterations The values correspond to samples taken from each individual autoclave, into which the batch of synthesis gel was divided.

**Autoclave No.**	***a* (Å)**	**SD**	***c* (Å)**	**SD**	**R_{wp} (%)**	**R_p**	**GoF**	**R_{exp}**
**1**	13.68148	0.00195	8.374977	0.00170	10.36433	8.099217	1.579938	6.559962
**2**	13.68478		8.37483		8.198697	6.422040	1.424682	5.754754
**3**	13.68623		8.376366		7.75492	6.029254	1.377008	5.631716
**4**	13.68581		8.371544		7.876865	6.221274	1.394296	5.649350
**5**	13.68620		8.375524		8.011889	6.284360	1.380451	5.803818
**6**	13.68683		8.375711		8.179998	6.424026	1.472615	5.554743
**AVG**	13.68524		8.374825		8.397784	6.580029	1.438165	5.825724

Table S 9 Rietveld refinement parameters

**Parameter**	**Value**
Refinement Software	*TOPAS*[63–65]
Radiation Source	Cu Kα (λ = 1.5406 Å)
Sample Geometry	Bragg Brentano (adhered to Si (9 1 1) slide with petroleum jelly)
Data Collection	Bruker D8 Advance (lab) diffractometer
Space Group	P6cc
Unit cell parameters	See Tables S5-8
R_{wp}	
R_p	
GoF	
R_{exp}	
Background model	Chebyshev polynomial (6 terms)
Peak Profile Function	Thompson-Cox-Hastings pseudo-voigt (TCHZ)
Structural Model	Entry ICSD-91671 from "Absolute configuration and domain structure of $AlPO_4$-5 studied by single crystal X-ray diffraction"[66]

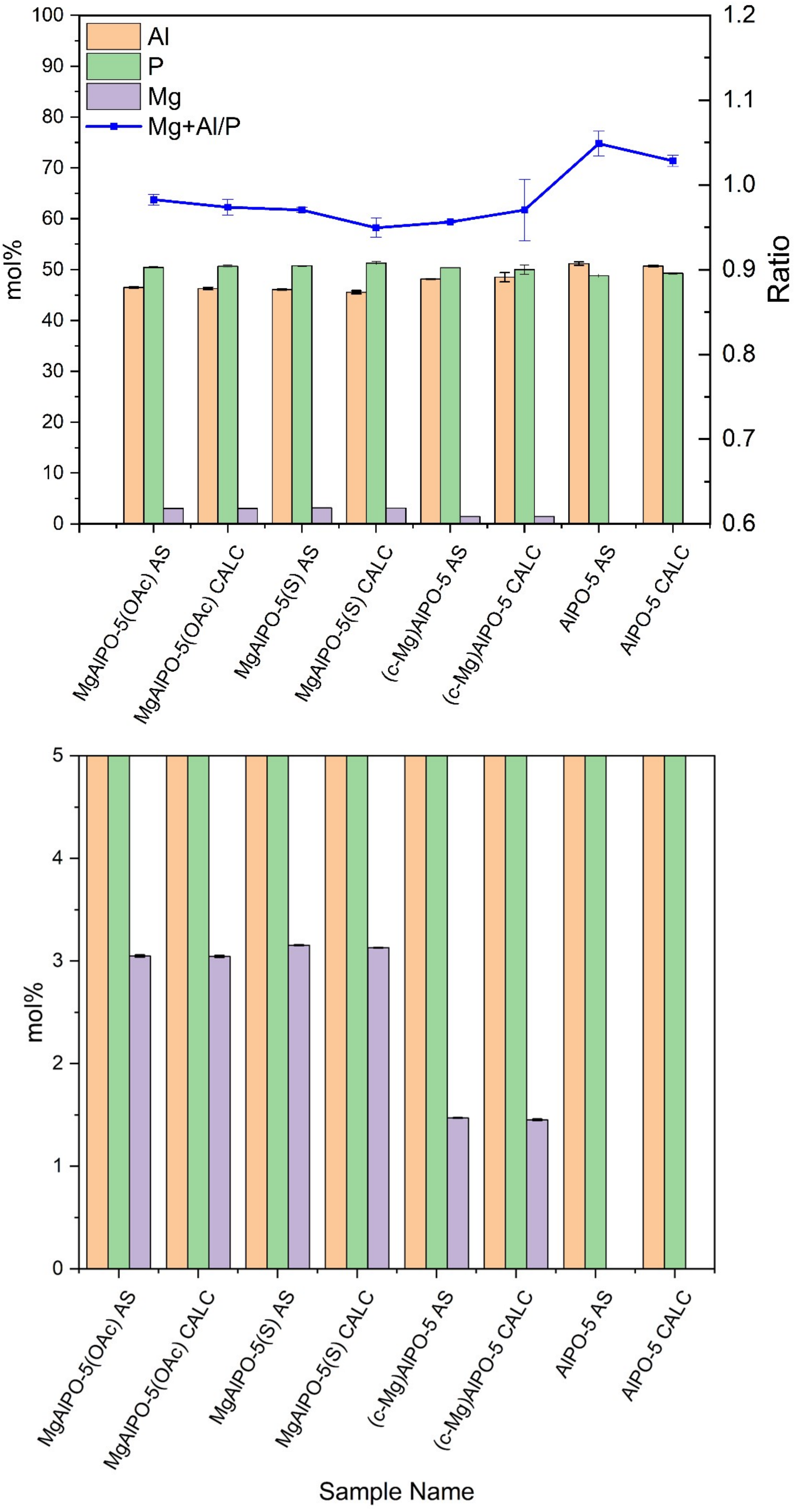

Figure S 8 Top - elemental analysis showing the amount of Al, P, and Mg (mol%) in each sample. The blue line shows the (Mg +Al)/P ratio. Error bars represent standard deviation calculated from triplicate analysis of the same sample. If not seen, then error is within the symbol. Bottom – enlarged as above to display Mg (mol%) more clearly. Mol% was calculated as mol of either Al, P or Mg, divided by the sum of all three (multiplied by 100).

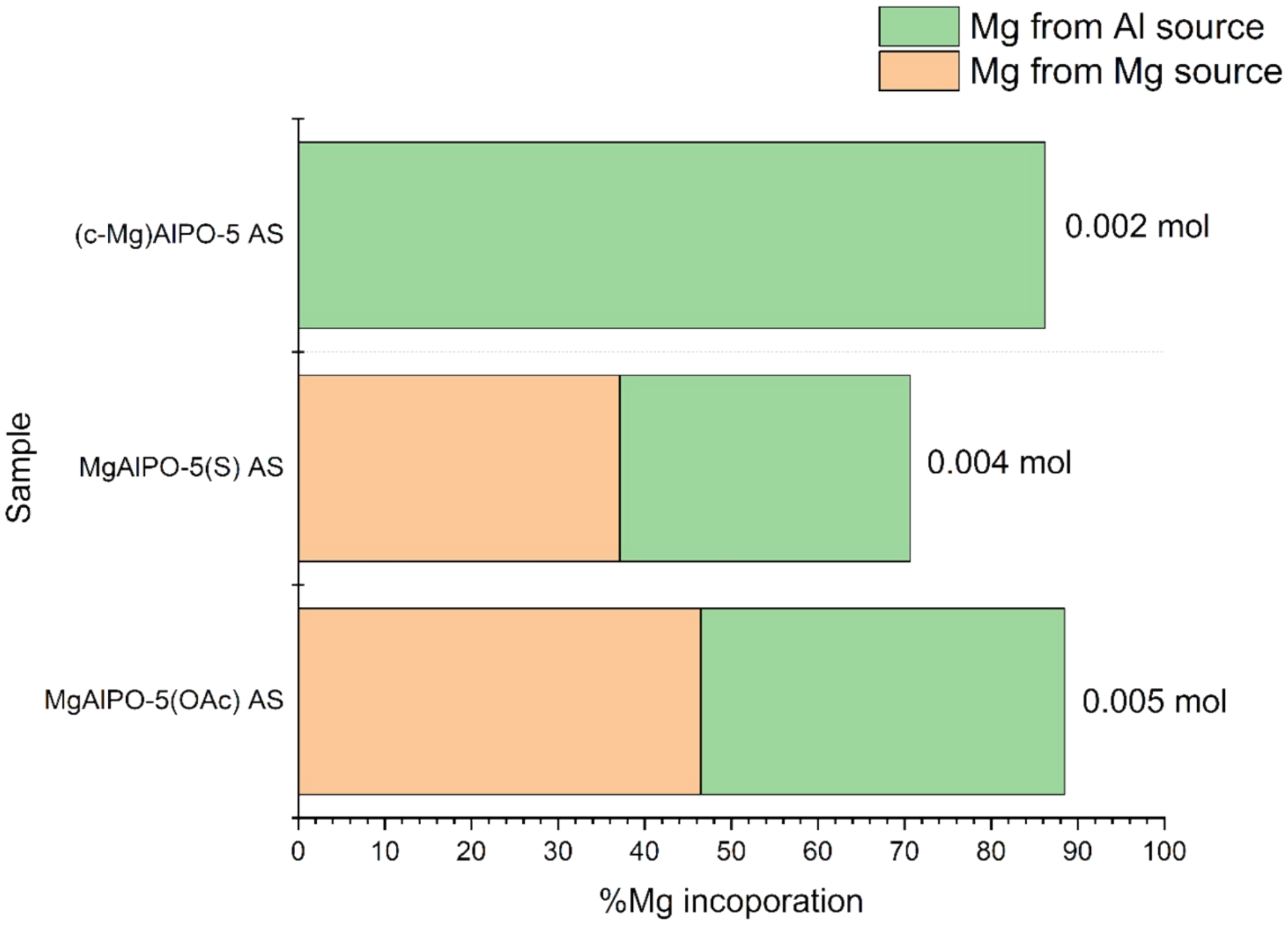

Figure S 9 %Mg incorporation showing calculated contributions from both the intended magnesium source (magnesium acetate or struvite) and magnesium present in the aluminium source ($Al(OH)_3{\cdot}xH_2O$). For both MgAlPO-5(OAc) and MgAlPO-5(S) each source contributed approximately 50%. The total %Mg incorporation was determined by dividing the Mg(mol) in the final AS product by Mg(mol) initially added to the gel. The contribution from each source (the Al source and the intended Mg source) was then calculated by multiplying the total %Mg incorporation by the fractional contribution of the Mg source to the total added to the gel. However, since %Mg incorporation was not 100% this calculation provides only an estimate of relative contributions and do not definitively represent the exact source distribution of Mg in the final product.

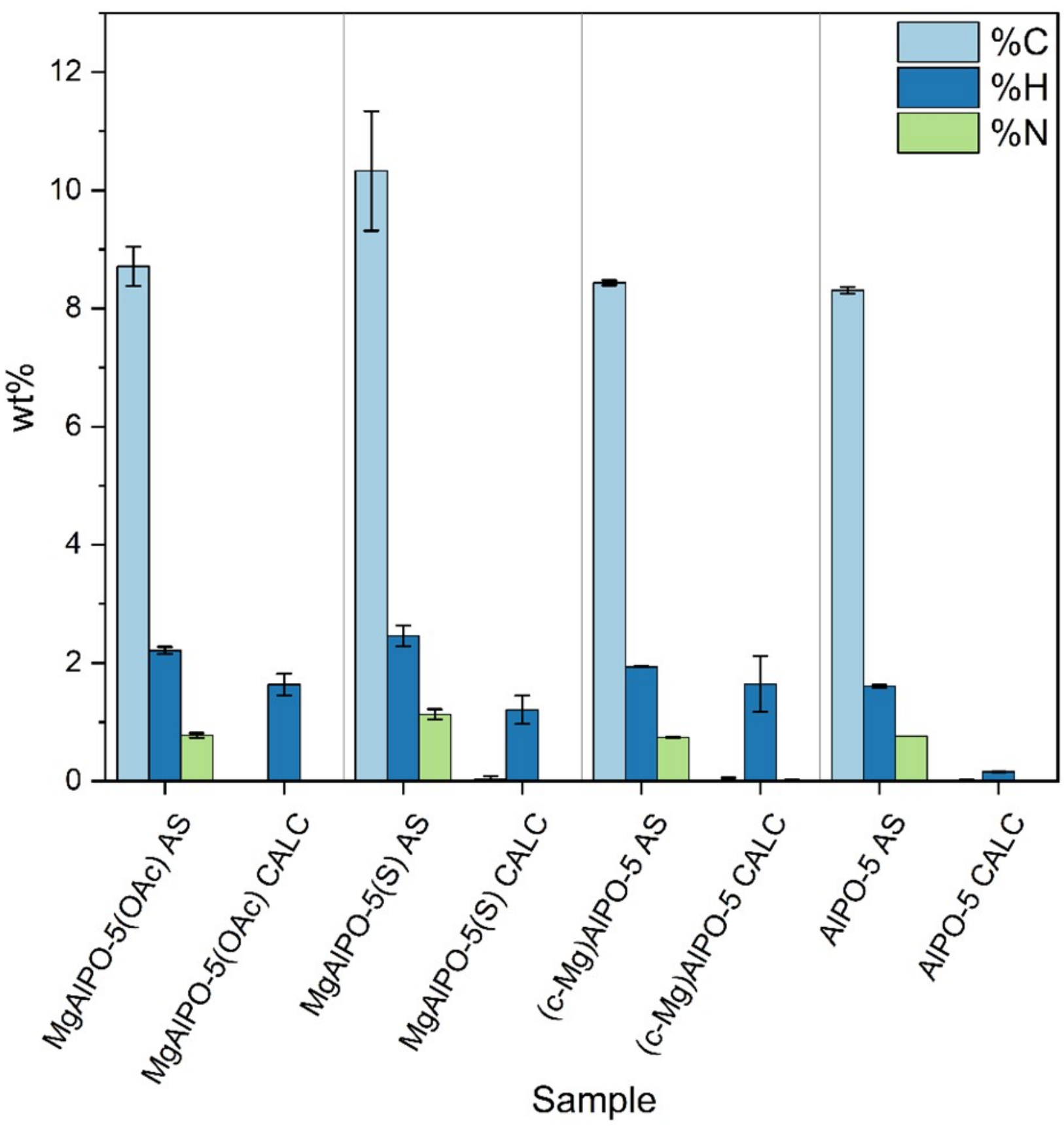

Figure S 10 CHN data for all samples as-synthesised and calcined, to show the successful removal of the N,N-dicyclohexylmethylamine (MDCHA) template by calcination. This can be seen by the significantly reduced C and N wt% in the calcined sample. Error bars represent standard deviation from triplicate analysis conducted on the same sample.

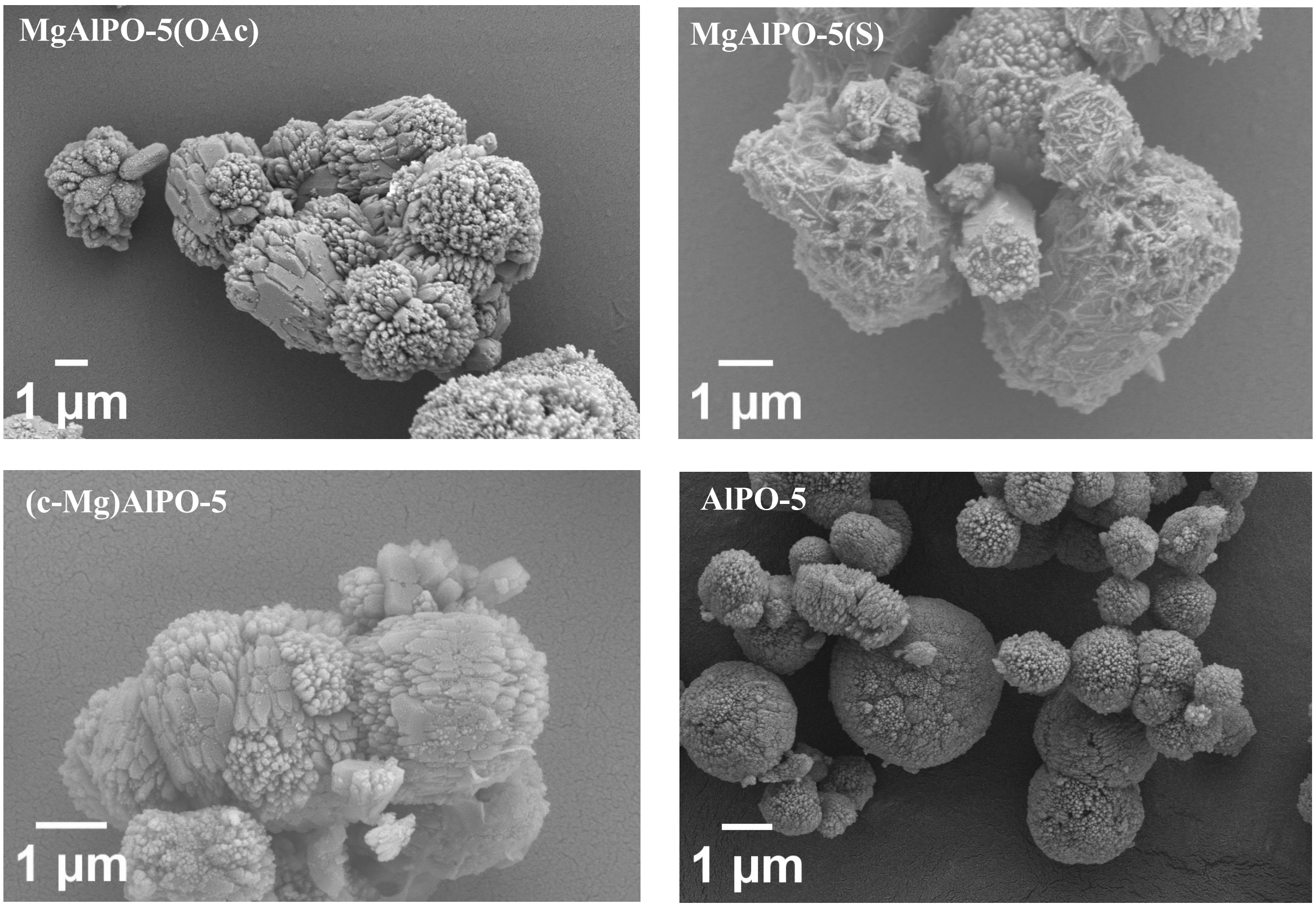

Figure S 11 SEM micrographs of all as-synthesised (AS) samples. The AlPO-5 has a visibly more rounded morphology when compared to those containing magnesium. Morphology is maintained upon calcination; particles seen here do not differ from calcined particles seen in Fig. 2b (main)

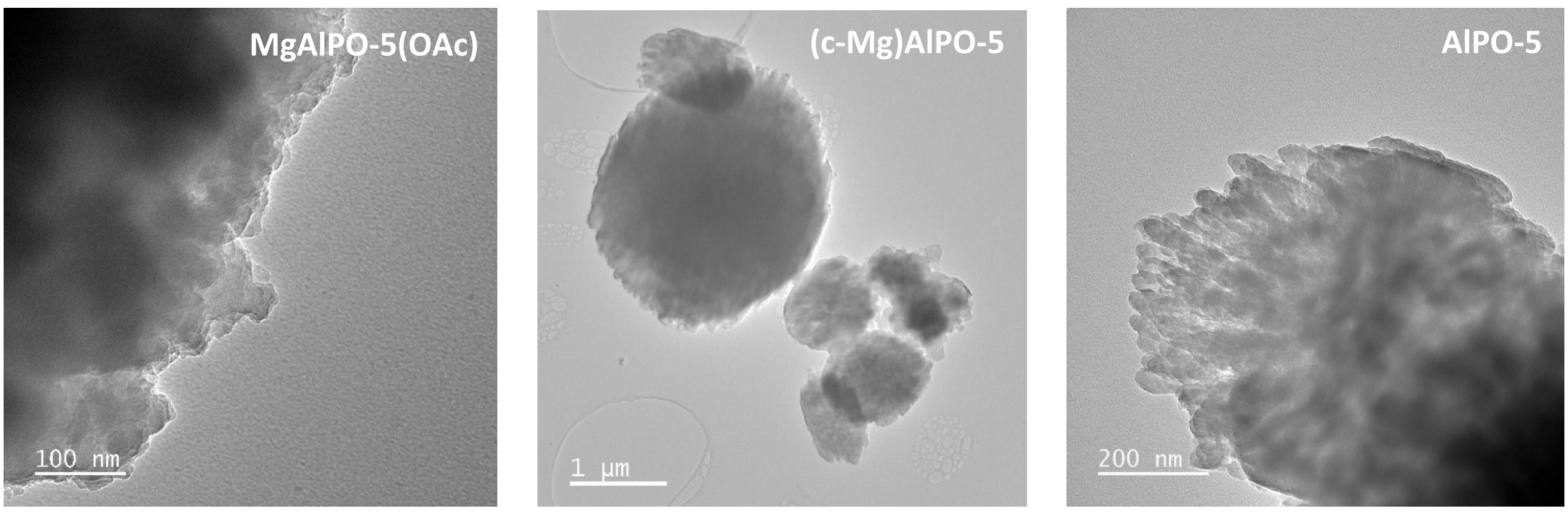

Figure S 12 TEM images of left: MgAlPO-5(OAc), centre: (c-Mg)AlPO-5 and right: AlPO-5 demonstrating the smoother exterior, not covered in nanosized crystallites as seen in MgAlPO-5(S) (Fig. 2c, main). All images are of calcined samples.

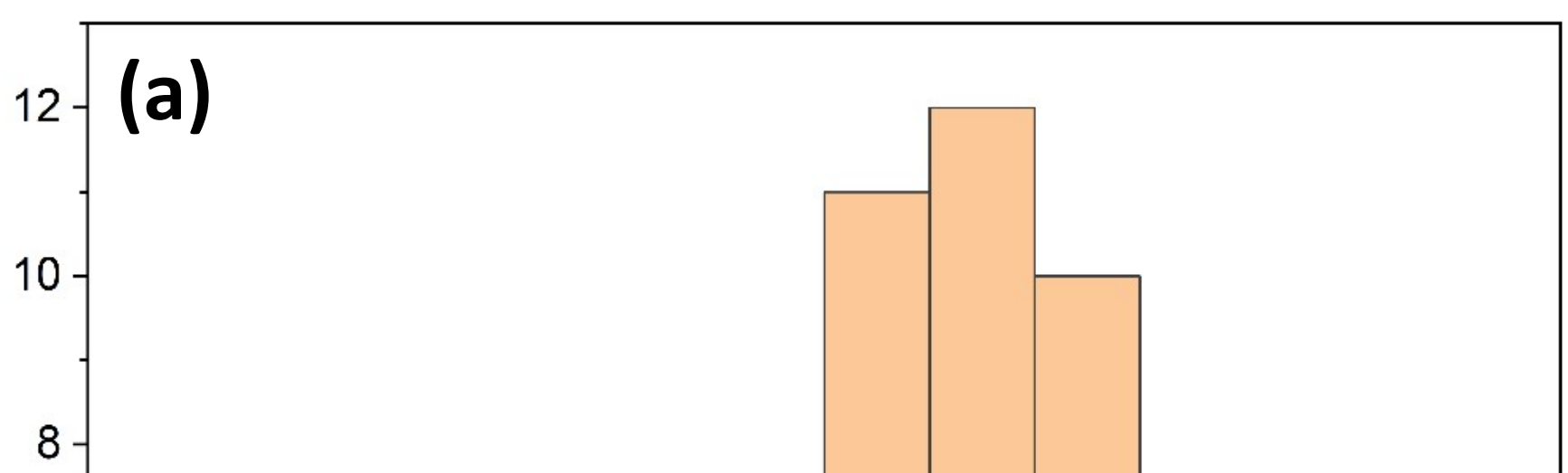

Figure S 13 (a) A histogram showing the widths of the surface nanogrowths on MgAlPO-5(S) measured from TEM images above. The median width is between 34 – 36 nm (34.314 nm) and the widths have a range of 18.134 nm. The sample size was 47; (b-c) TEM images annotated with lines, and measurements (in nm). This was performed using Image J (Fiji).

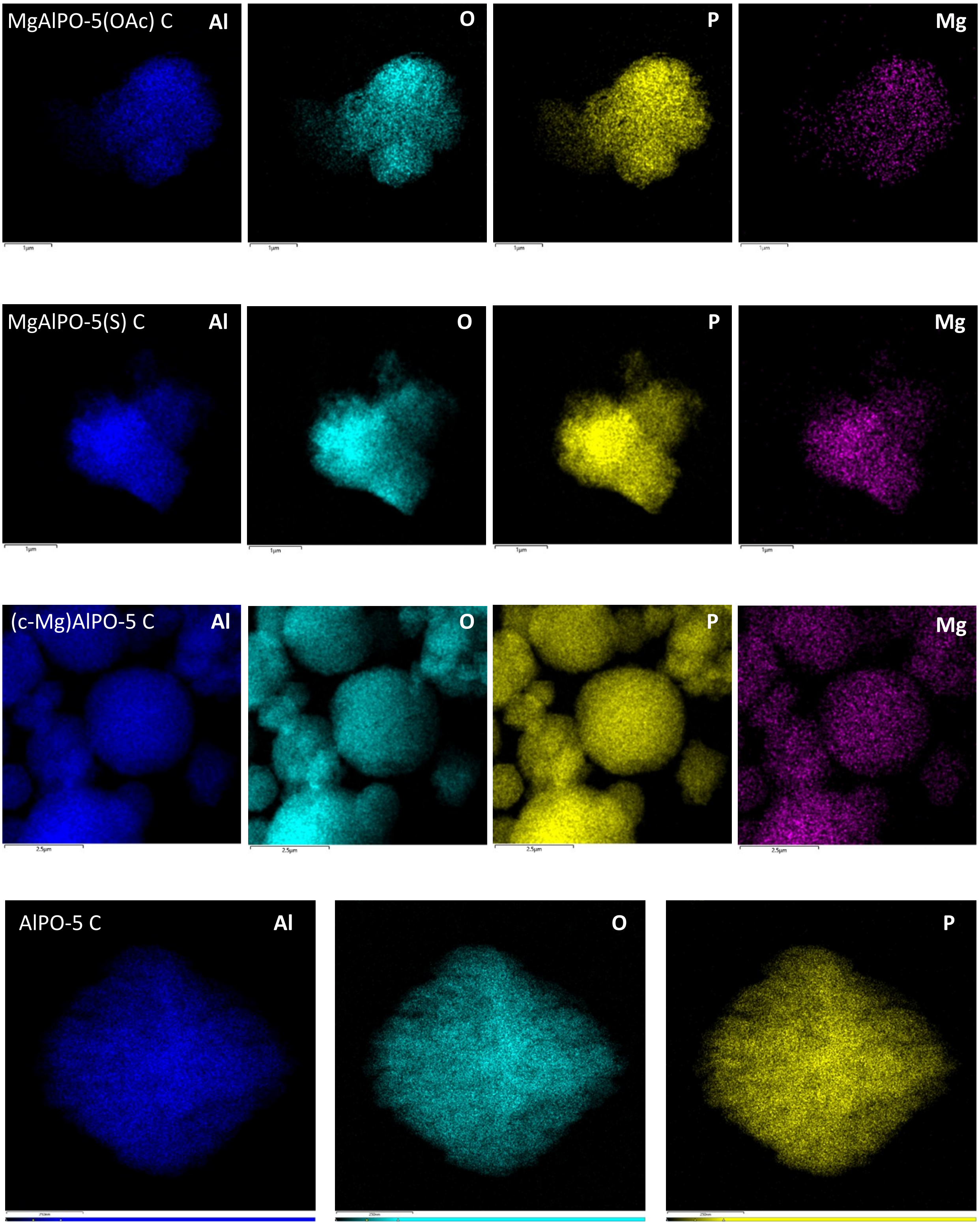

Figure S 14 TEM-EDS images showing chemical maps of Al, P, O and Mg for all of the calcined samples, from top to bottom: MgAlPO-5(OAc), MgAlPO-5(S), (c-Mg)AlPO-5 and AlPO-5.

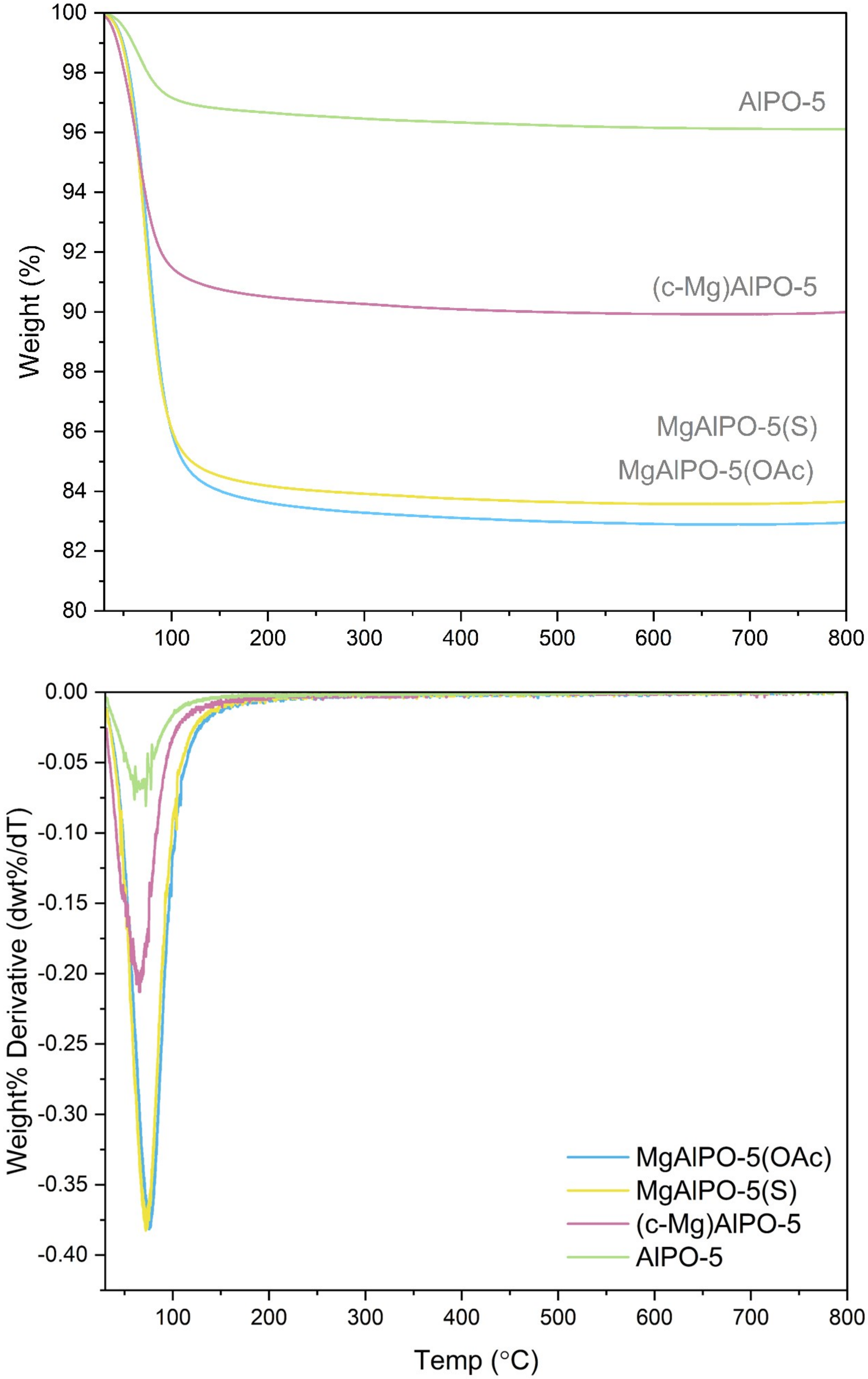

Figure S 15 Top - TGA traces of all four samples (calcined) heated from 30 – 800 °C. AlPO-5 shows the smallest mass loss, followed by (c-Mg)AlPO-5 and lastly both MgAlPO-5(OAc) and MgAlPO-5(S) which show similar mass losses. Bottom – DTG curve showing clearly the onset temperature of each mass loss event, which all occur between 65 – 75 °C.

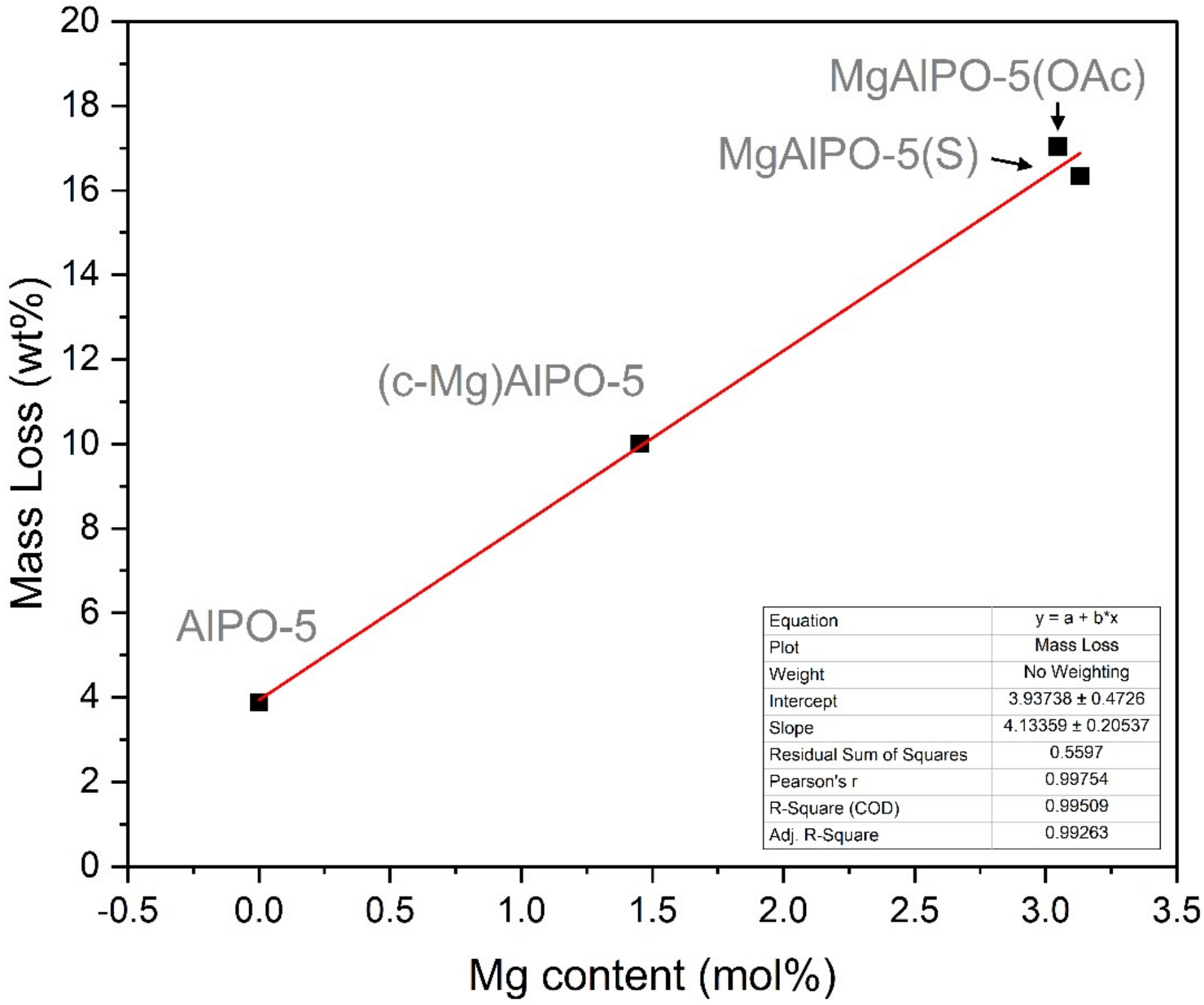

Figure S 16 Illustration of the Mg content (mol%) of each sample against the total mass loss recorded by TGA at 800 °C. A linear trendline was fitted to the data, yielding an R^2 value of 0.99509, indicating a strong correlation between the magnesium content within the framework and the mass loss measured by TGA.

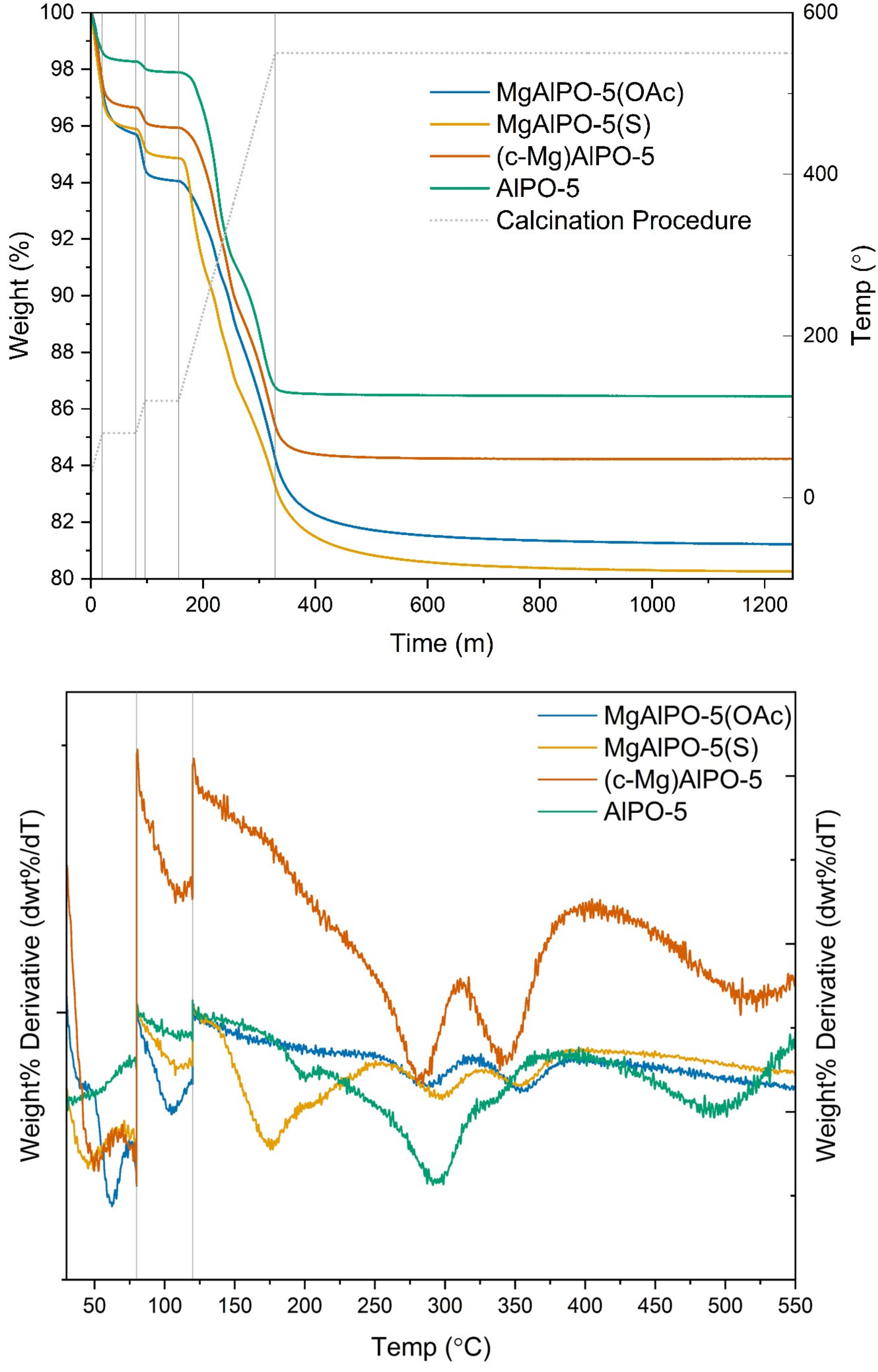

Figure S 17 Top – TGA traces of the calcination protocol with respect to time (80 °C – 1 h – 120 °C – 1h – 550 ° - 16 h) with grey vertical lines denoting each stage of the process. The calcination ramp is shown as a grey dotted line and refers to the time and temperature (y2 axis). Bottom – DTG composed of 3 sections; ramp to 80 °C, 1 h hold; ramp 120 °C, 1 h hold; and ramp to 550 °C, 16 h hold. Hold sections are shown by the grey vertical lines. The (c-Mg)AlPO-5 sample is plotted on the right-hand y-axis.

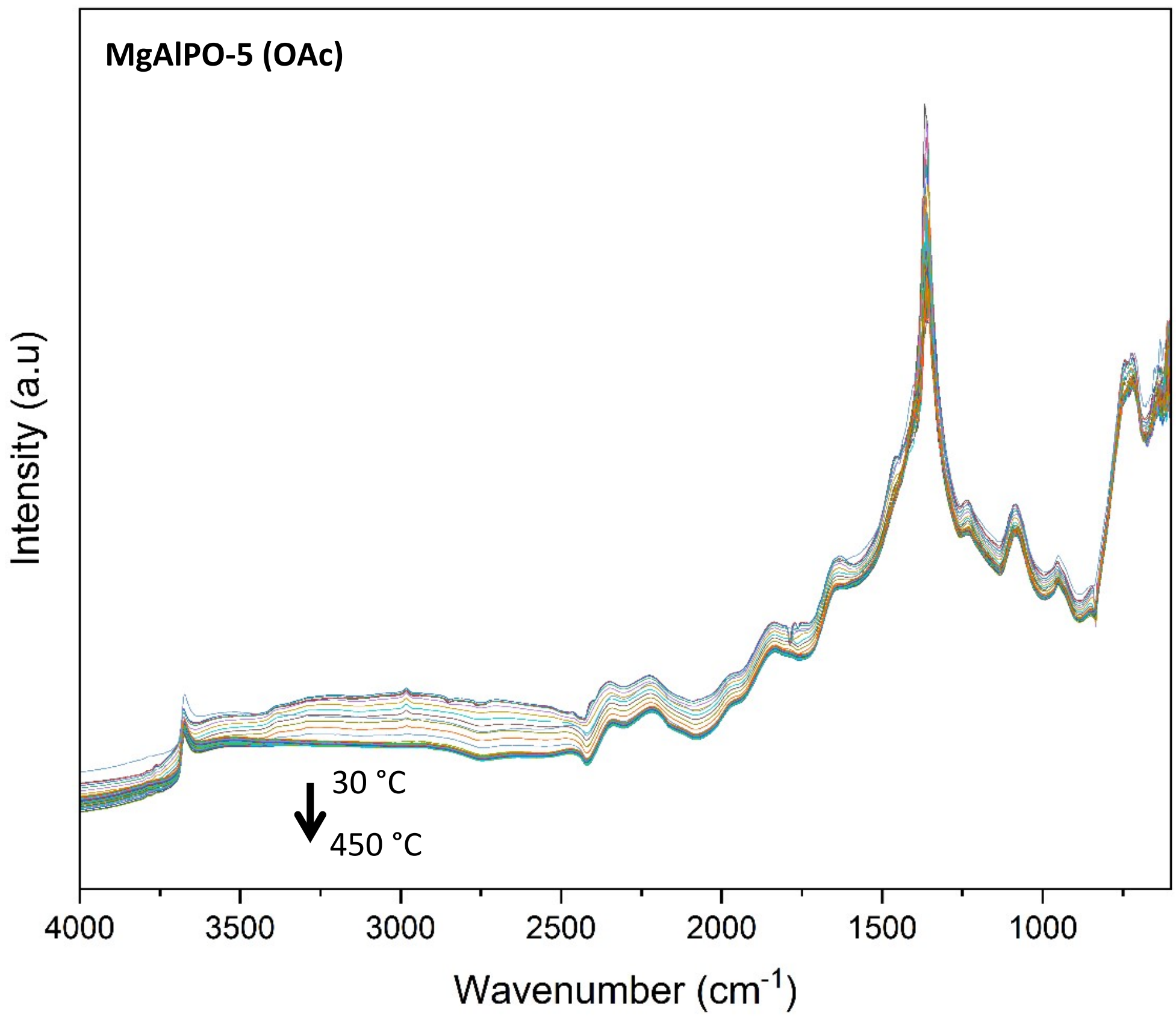

Figure S 18 The DRIFTS spectra for MgAlPO-5(OAc) were recorded throughout the entire temperature ramp from 30 to 450 °C. The arrow indicates the direction of increasing temperature, with spectra at the bottom corresponding to higher temperatures than those at the top.

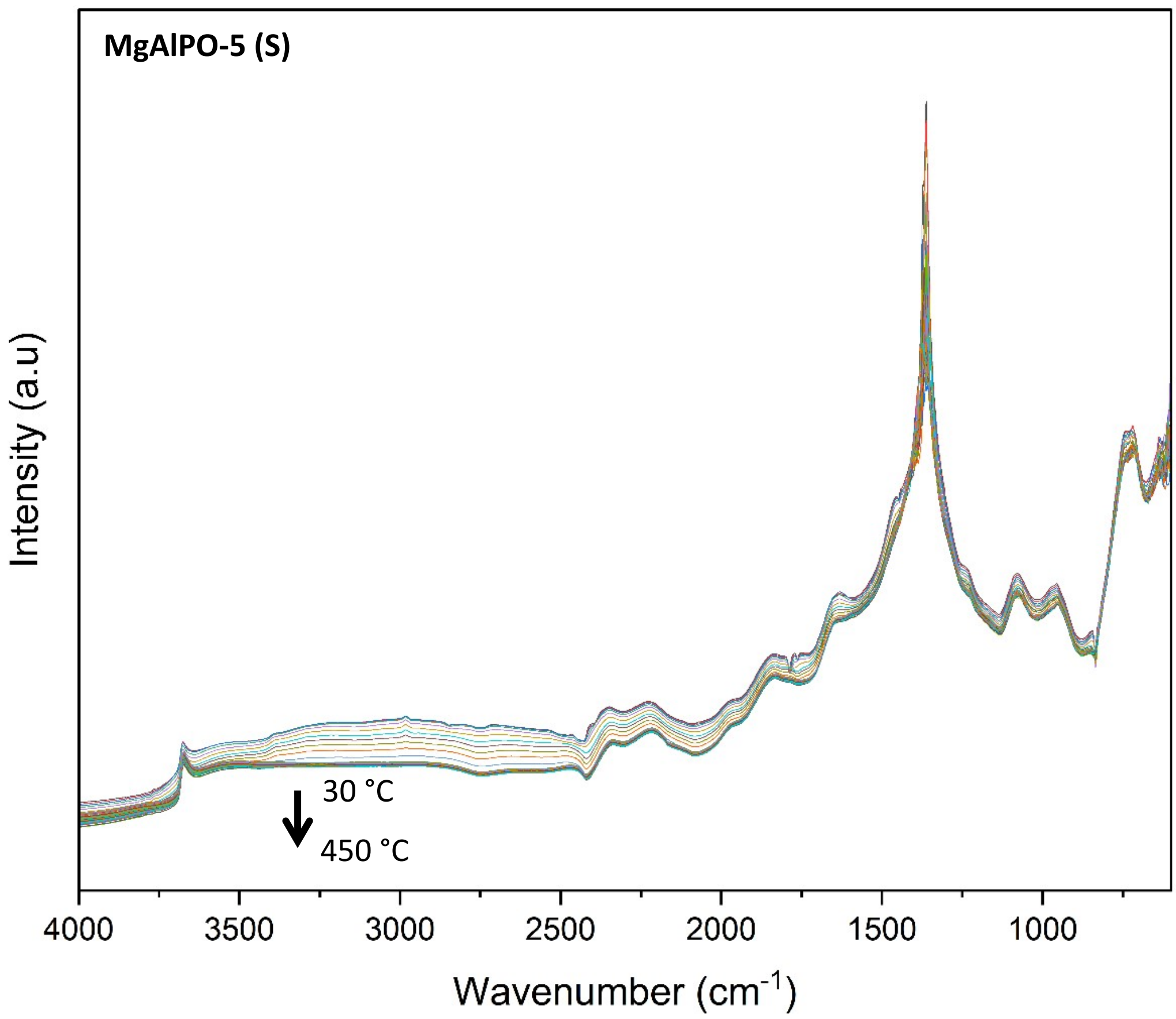

Figure S 19 DRIFTS spectra for MgAlPO-5(S); refer to Fig. S18 caption for experimental details.

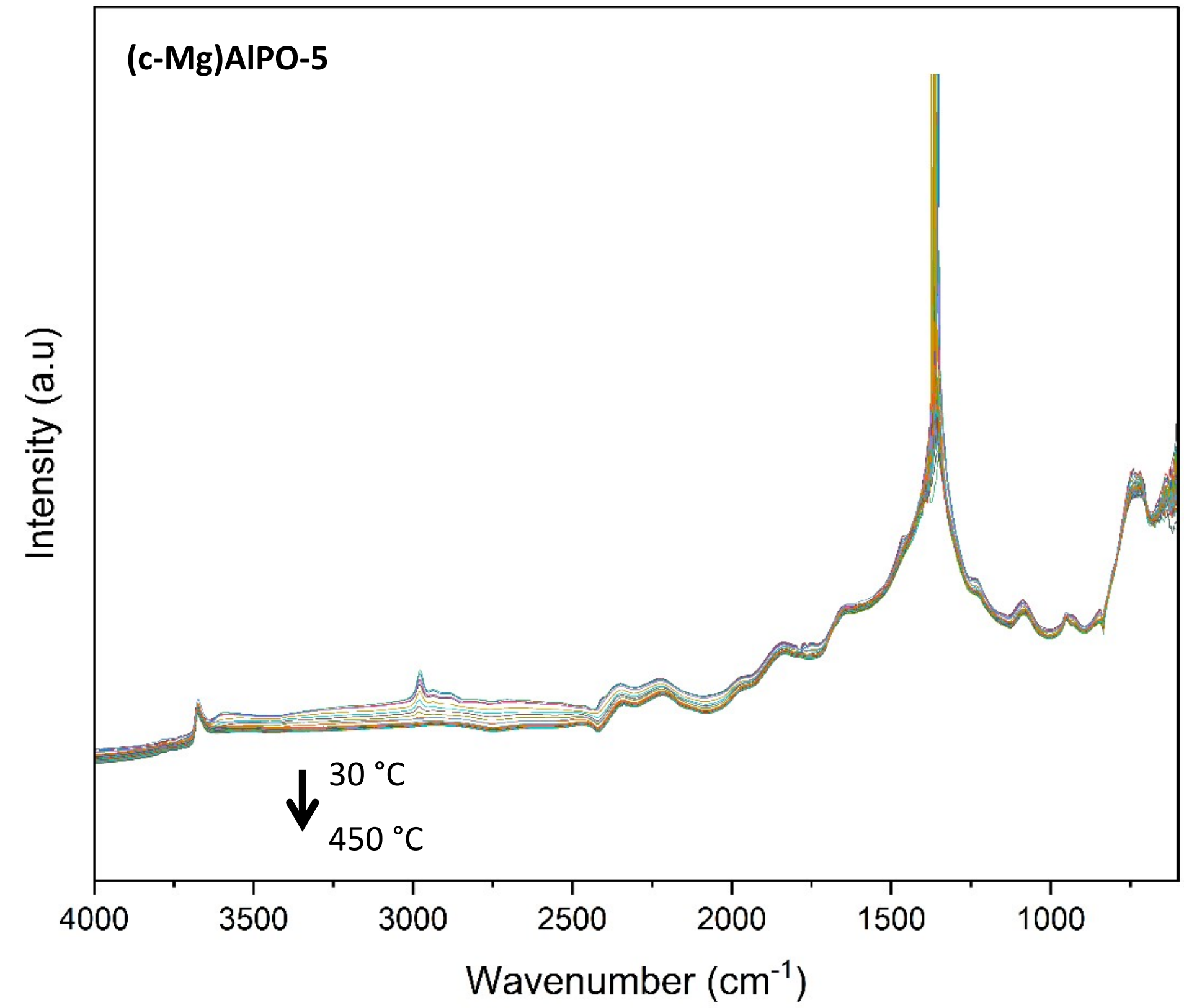

Figure S 20 DRIFTS spectra for (c-Mg)AlPO-5; refer to Fig. S18 caption for experimental details.

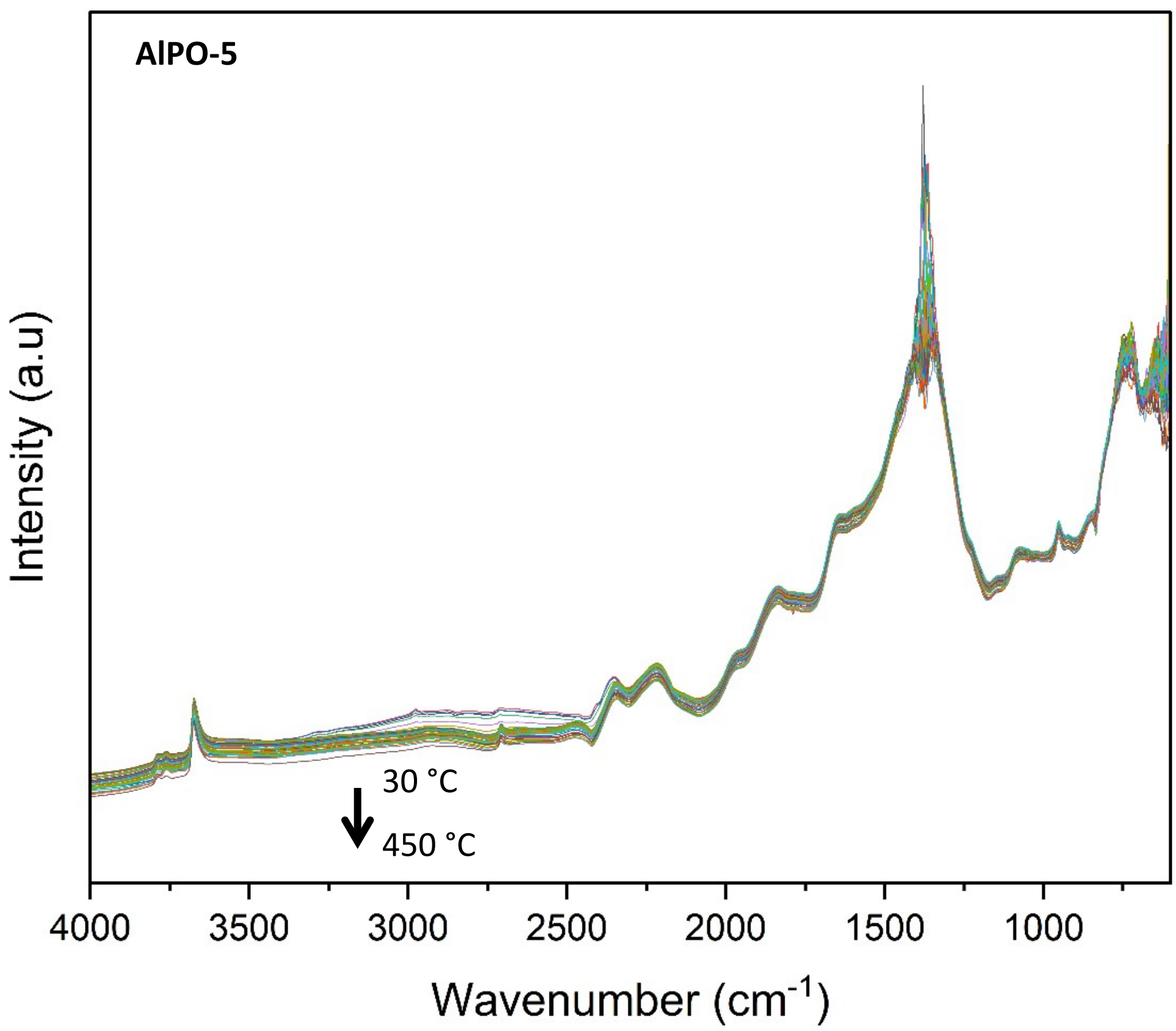

Figure S 21 DRIFTS spectra for AlPO-5; refer to Fig. S18 caption for experimental details.

A thermocouple data logger has been used to monitor the temperature of the sample bed being measured by a thermocouple inserted into the Harrick Praying Mantis™ high temperature reaction chamber on the DRIFTS set-up (see pg 4 – Full Characterisation Details). Discrepancies between the temperature of the sample bed and Harrick ATK controlled heat element have been previously reported.[2] Here the heater was set to 500 °C however the sample bed only reached ~450 °C.

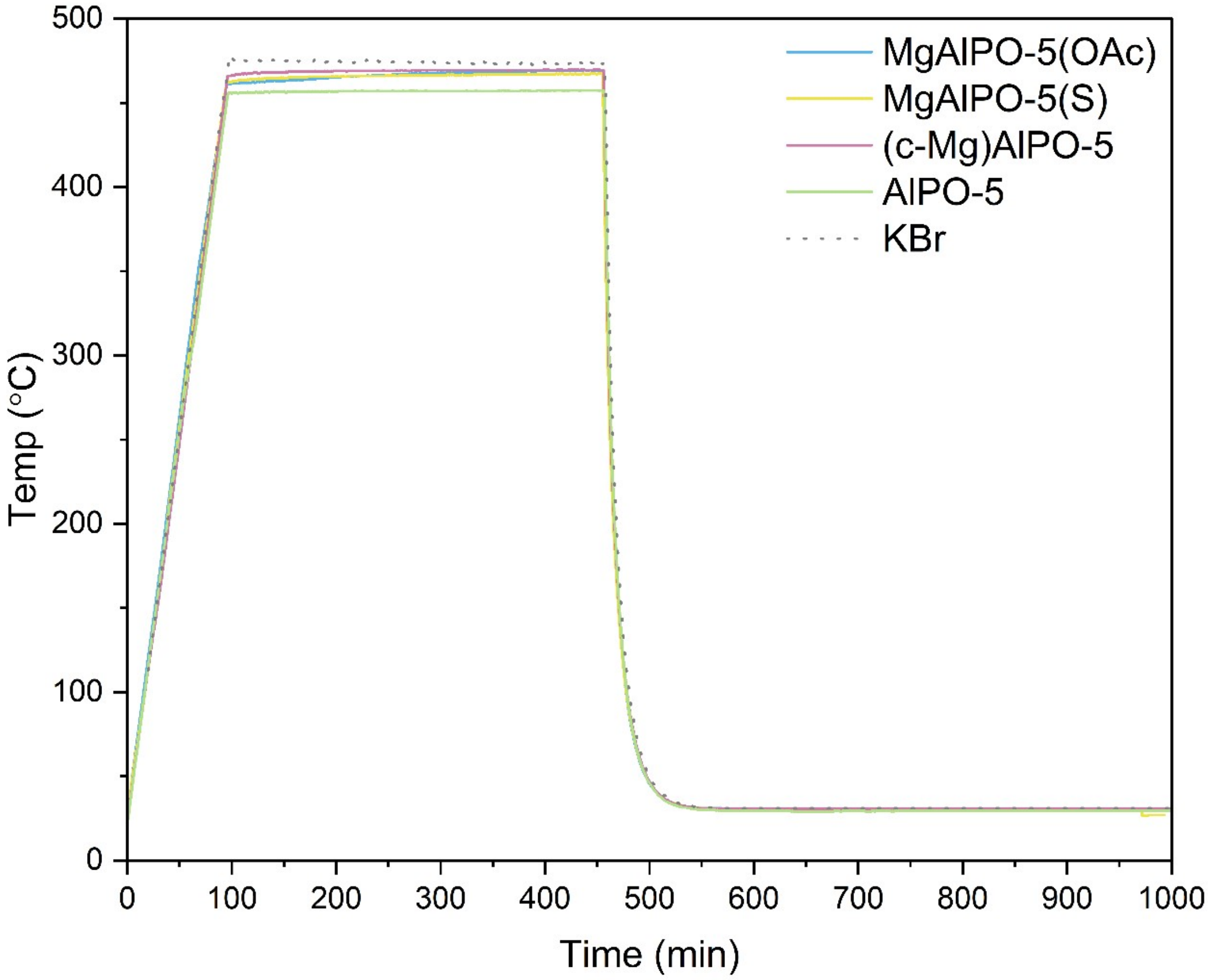

Figure S 22 The temperature of the samples, measured using a thermocouple couple inserted through the sample holder wall, connected to an external data logger. Temperature discrepancies between the cartridge heater (500 °C here), and actual sample temperature (~450 – 480 °C) are a well-documented problem associated with the Harrick cell.

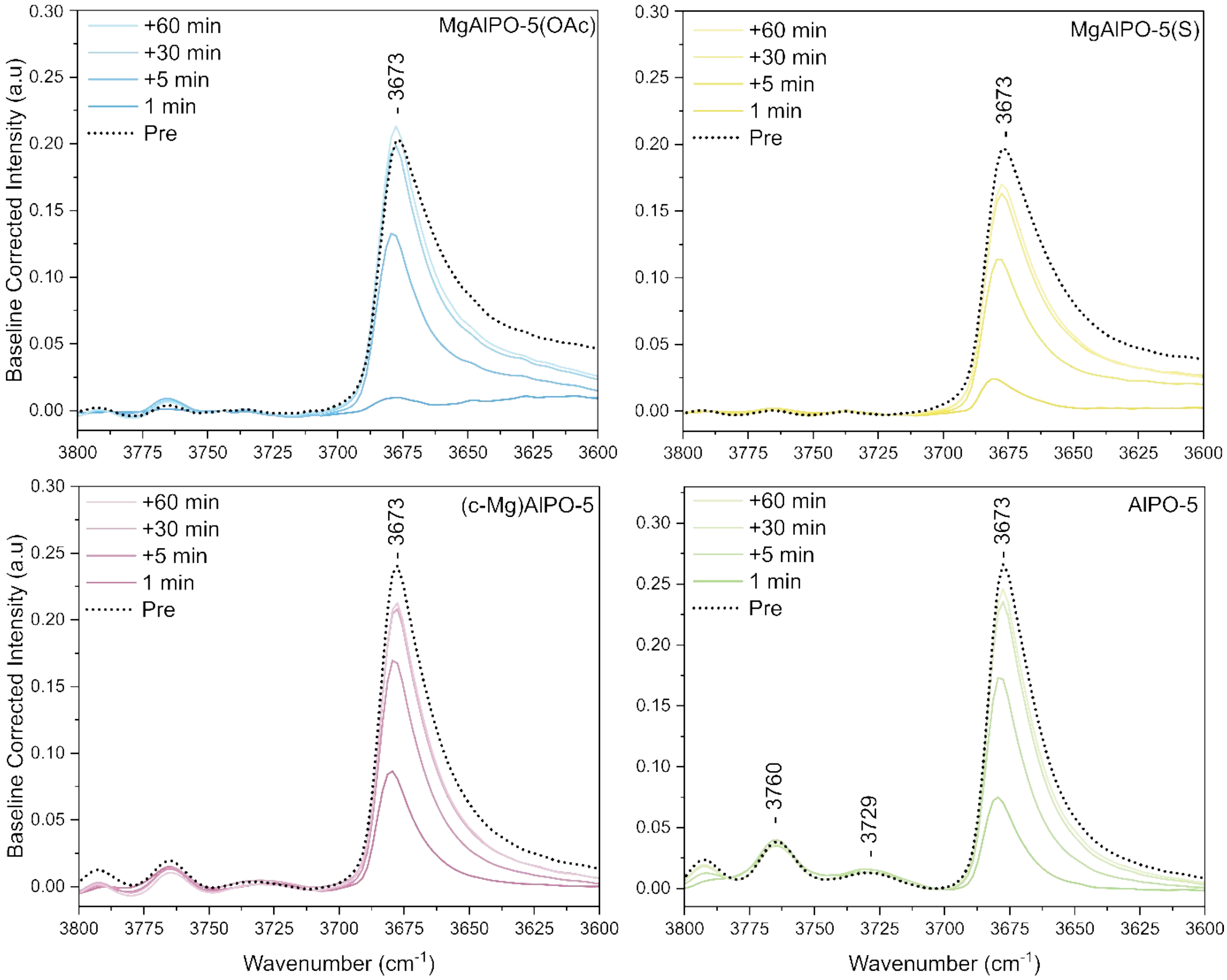

Figure S 23 Baseline corrected DRIFTS spectra using a CD_3CN probe. The black dotted line shows the framework without acetonitrile. The increasingly light spectra show increasing time from the addition of CD_3CN into the DRIFTS cell: 1 min = immediately after the sample has been exposed to CD_3CN for 1 min; + 5 min = dynamic vacuum up to ~10^{-1} hPa 5 min post CD_3CN addition; + 30 min = dynamic vacuum increased to ~10^{-2} – 10^{-3} hPa post CD_3CN addition; +60 min = dynamic vacuum between ~10^{-2} – 10^{-3} hPa post CD_3CN addition for an additional 30 min, totalling 60 min. Peaks potentially attributed to Al-OH groups can be most clearly seen in the AlPO-5 spectra (bottom right) at $\tilde{\nu}$ = 3760 and 3729 cm^{-1}. It would be expected (as with the P-OH peak at $\tilde{\nu}$ = 3675 cm^{-1}) that these peaks decrease in intensity upon CD_3CN addition, but this is not the case, therefore the assignment of the peaks remains uncertain.

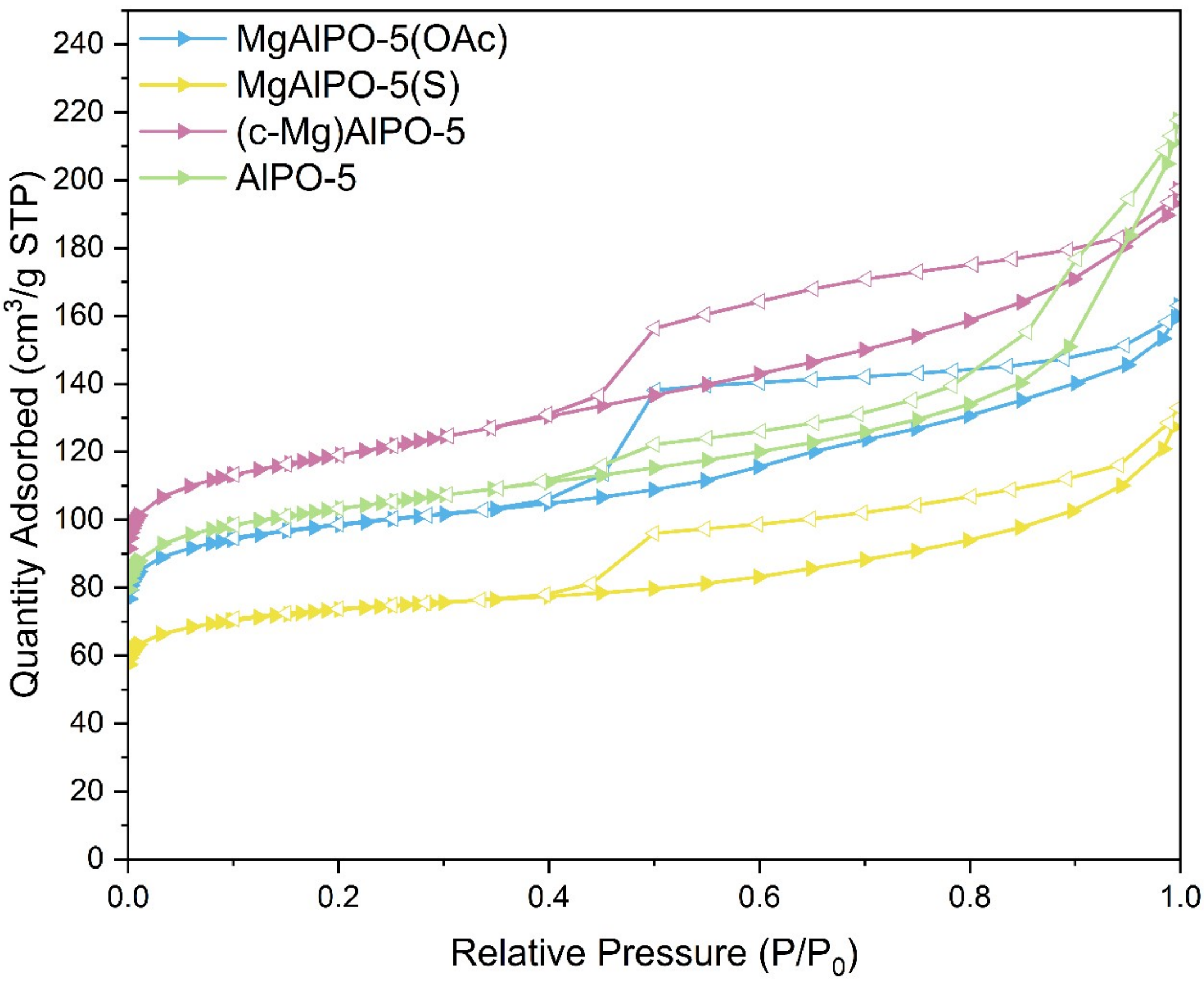

Figure S 24 Nitrogen isotherms for all calcined materials.

Table S 10 BET areas determined from the nitrogen isotherms in a range of p/p_0 = 0.01 – 0.1. Micropore area, external surface area and micropore volume determined from the t-plot method.

**Sample**	**BET Surface Area (m^2/g)**	**t-plot Micropore Area (m^2/g)**	**t-plot External Surface Area (m^2/g)**	**t-plot Micropore Volume (cm^3/g)**
MgAlPO-5(OAc)	377	281	90	0.11
MgAlPO-5(S)	281	213	65	0.09
(c-Mg)AlPO-5	453	316	130	0.13
AlPO-5	394	285	104	0.11

Table S 11 The measured rate of ethylene production (mmolmin^{-1}) in the acid-catalysed dehydration of ethanol is shown below. Peak area for ethylene as seen in the BID, and calculated mmolmin^{-1} for samples MgAlPO-5(OAc), MgAlPO-5(S) and AlPO-5. The onset of ethylene production is highlighted in yellow. Continues to next page.

Sample	Temp (°C)	Time at Temp (min)	Ethylene Peak Area	Rate of Ethylene Production (mmolmin^{-1})	Rate of Ethylene Production per Gram of Catalyst (mmol min^{-1} g^{-1})	Rate of Ethylene Production per moles of Mg (mmol min^{-1} Mg^{-1})
MgAlPO-5 (OAc)	150	0	0	0		
		30	0	0		
		60	0	0		
		90	0	0		
	200	0	0	0		
		30	0	0		
		60	913578	0.00571	0.01903	1.05741
		90	931013	0.00582	0.01940	1.07778

Sample	Temp (°C)	Time at Temp (min)	Ethylene Peak Area	Rate of Ethylene Production (mmolmin^{-1})	Rate of Ethylene Production per Gram of Catalyst (mmol min^{-1} g^{-1})	Rate of Ethylene Production per moles of Mg (mmol min^{-1} Mg^{-1})
MgAlPO-5 (S)	150	0	0	0		
		30	0	0		
		60	0	0		
		90	0	0		
	200	0	0	0		
		30	0	0		
		60	512130	0.00320	0.01067	0.55440
		90	449624	0.00281	0.00937	0.48683

Sample	Temp/ °C	Time at Temp (min)	Ethylene Peak Area	Rate of Ethylene Production (mmol min^{-1})	Rate of Ethylene Production per Gram of Catalyst (mmol min^{-1} g^{-1})	Rate of Ethylene Production per moles of Mg (mmol min^{-1} Mg^{-1})
AlPO-5	150	0	0	0		
		30	0	0		
		60	0	0		
		90	0	0		
	200	0	0	0		
		30	0	0		
		60	99194	0.00062	0.00207	N/A
		90	87714	0.00055	0.00183	

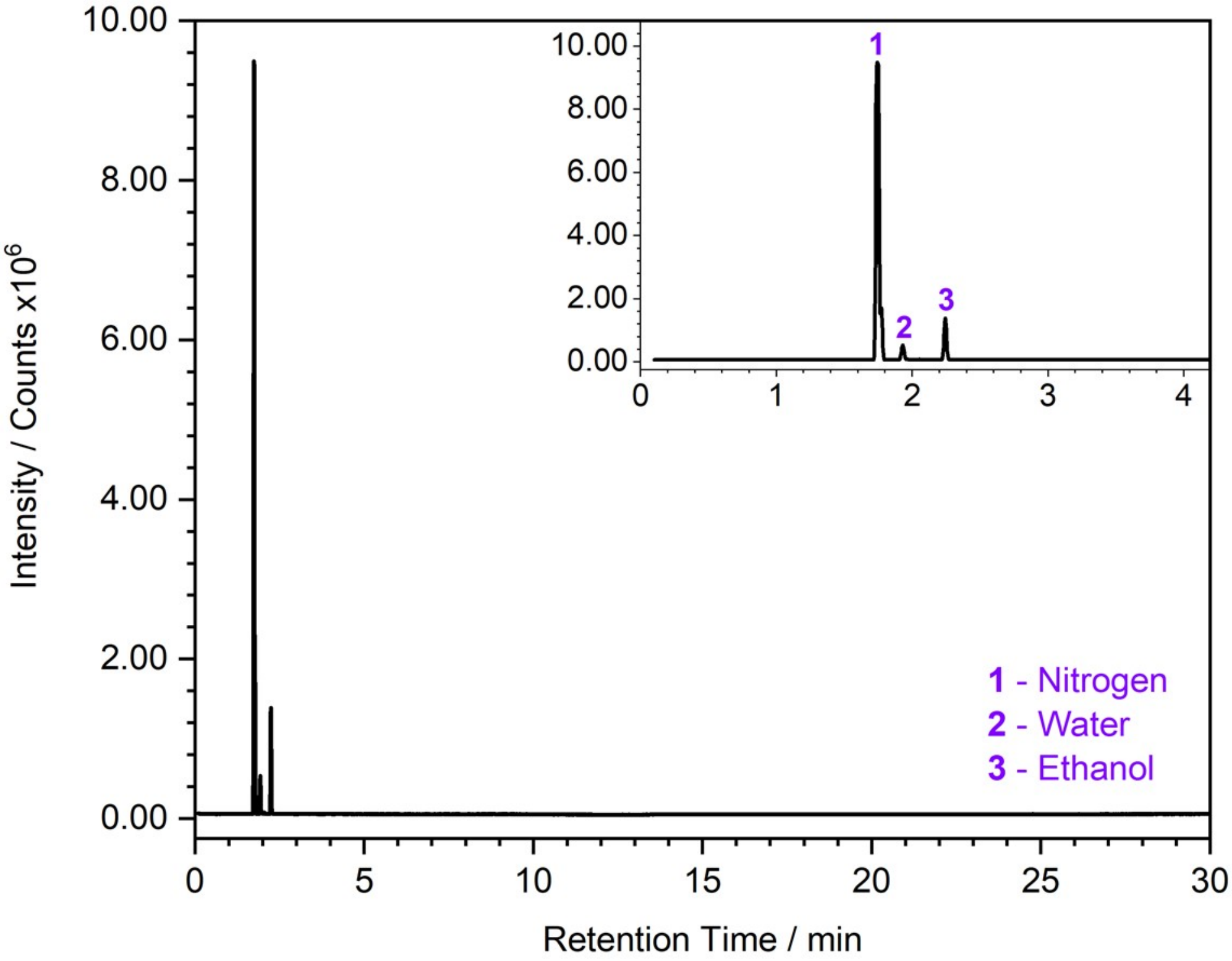

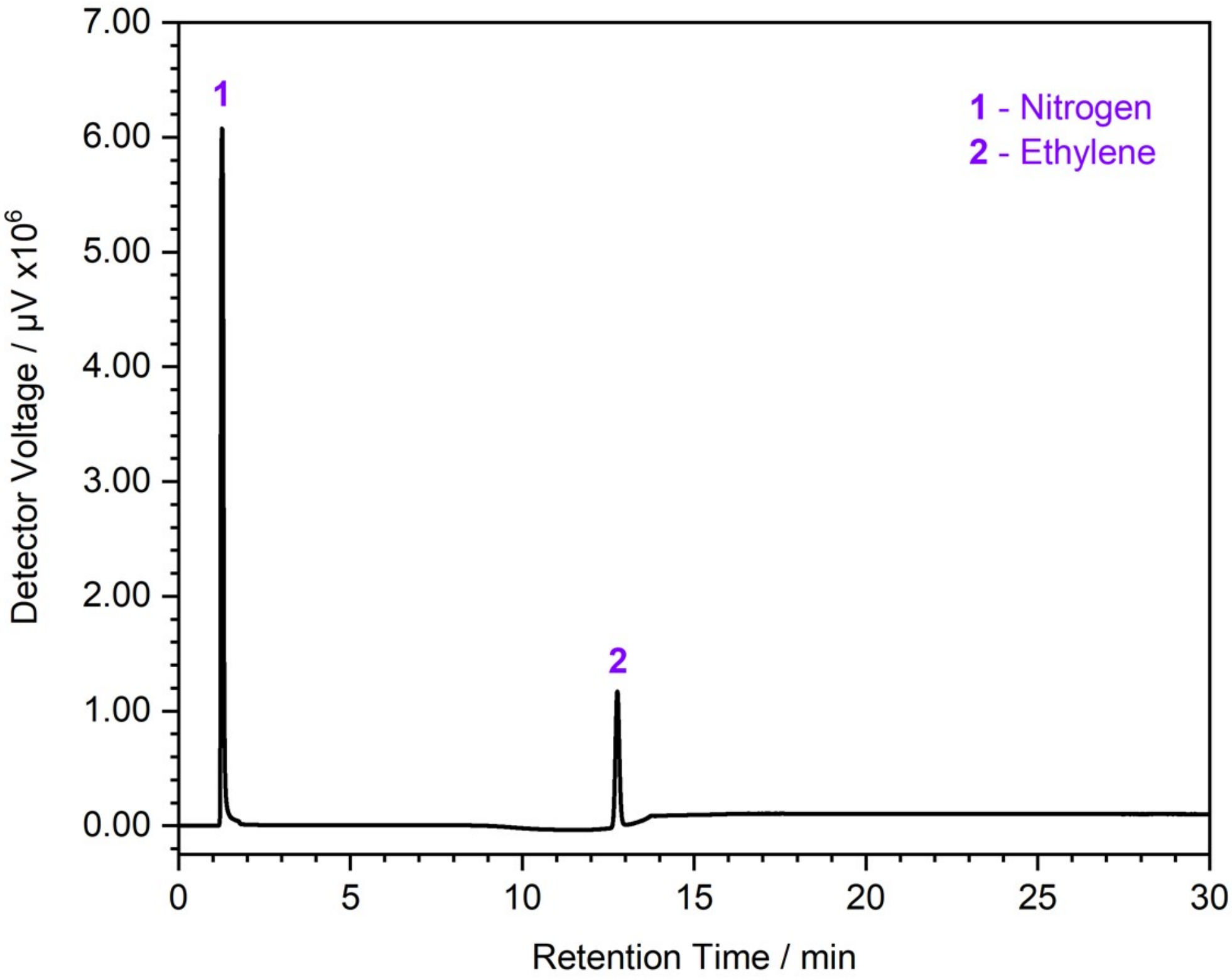

Figure S 25 Example MS (top) and BID (bottom) trace resulting from an ethanol to ethylene reaction over AlPO-5, recorded at 200 °C with a TOS of 1 h. Detection columns used were an RTX-VMS (MS) and a ShinCarbon ST (BID).

References

1 M. Sanchez-Sanchez, A. Simperler, R. G. Bell, C. Richard, A. Catlow and J. M. Thomas, The extremely high specificity of N-methyldicyclohexylamine for the production of the large-pore microporous AFI material, *Catal Letters*, 2003, **88**, 163–167.

2 H. Li, M. Rivallan, F. Thibault-Starzyk, A. Travert and F. C. Meunier, Effective bulk and surface temperatures of the catalyst bed of FT-IR cells used for in situ and operando studies, *Physical Chemistry Chemical Physics*, 2013, **15**, 7321.

3 S. L. Cresswell, J. R. Parsonage, P. G. Riby and M. J. K. Thomas, Rapid synthesis of magnesium aluminophosphate-5 by microwave dielectric heating, *J. Chem. Soc., Dalton Trans.*, 1995, 2315.

4 P. Concepción, J. M. L. Nieto, A. Mifsud and J. Pérez-Pariente, Preparation and characterization of Mg-containing AFI and chabazite-type materials, *Zeolites*, 1996, **16**, 56–64.

5 S. K. Saha, S. B. Waghmode, Y. Kubota and Y. Sugi, Synthesis of AFI aluminophosphate molecular sieves partly substituted with magnesium by dry-gel conversion method, *Mater Lett*, 2004, **58**, 2918–2923.

6 L. Feng, X. Qi, J. Li, Y. Zhu and L. Zhu, Synthesis of MgAPO-5 molecular sieves and their kinetic behavior for n-hexane cracking, *Reaction Kinetics and Catalysis Letters*, 2009, **98**, 327–339.

7 M. E. Potter, L. Spiske, P. N. Plessow, E. B. McShane, M. Carravetta, A. E. Oakley, T. Bere, J. H. Carter, B. D. Vandegehuchte, K. M. Kaźmierczak, F. Studt and R. Raja, Combining computational and experimental studies to gain mechanistic insights for *n* -butane isomerisation with a model microporous catalyst, *Catal. Sci. Technol.*, 2024, 7140–7151.

8 D. B. Akolekar, Acidity and catalytic properties of AlPO4-11, SAPO-11, MAPO-11, NiAPO-11, MnAPO-11 and MnAPSO-11 molecular sieves, *J Mol Catal A Chem*, 1995, **104**, 95–102.

9 R. Fernandez, M. V. Giotto, H. O. Pastore and D. Cardoso, Synthesis and characterization of MAPO-11 molecular sieves, *Microporous and Mesoporous Materials*, 2002, **53**, 135–144.

10 Y. Xu, Z. Tian, S. Wang, L. Wang, L. Hou, Y. Ma, Y. Wei, H. Ma, B. Wang, Z. Xu, J. Yu and L. Lin, 2007, pp. 228–232.

11 R. Sola-Llano, A. Oliden-Sánchez, A. Alfayate, L. Gómez-Hortigüela, J. Pérez-Pariente, T. Arbeloa, J. Hofkens, E. Fron and V. Martínez-Martínez, White Light Emission by Simultaneous One Pot Encapsulation of Dyes into One-Dimensional Channelled Aluminophosphate, *Nanomaterials*, 2020, **10**, 1173.

12 L. Zhang, D. S. Firth, U. Olsbye and X. Bao, Two-Step Dry Gel Method Produces MgAPO-11 with Low Aspect Ratio and Improved Catalytic Performance in the Conversion of Methanol to Hydrocarbons, *Catalysts*, 2022, **12**, 413.

13 M. J. Maple and C. D. Williams, Synthesis and characterisation of aluminophosphate-based zeotype materials prepared with α,ω-bis(N-methylpyrrolidinium)alkane cations as structure-directing agents, *Dalton Transactions*, 2007, 4175.

14 M. da S. Machado, D. Cardoso, J. Pérez-Pariente and E. Sastre, Synthesis and Characterization of $AlPO_4$-36 and MAPO-36 with Different Magnesium Content, *Chemistry of Materials*, 1999, **11**, 3238–3244.

15 J. Xu, D. Zhou, X. Song, L. Chen, J. Yu, C. Ye and F. Deng, Crystallization of magnesium substituted aluminophosphate of type-36 as studied by solid-state NMR spectroscopy, *Microporous and Mesoporous Materials*, 2008, **115**, 576–584.

16 R. García, V. Martínez-Martínez, R. Sola Llano, I. López-Arbeloa and J. Pérez-Pariente, One-Dimensional Antenna Systems by Crystallization Inclusion of Dyes (One-Pot Synthesis) within Zeolitic MgAPO-36 Nanochannels, *The Journal of Physical Chemistry C*, 2013, **117**, 24063–24070.

17 M. G. Ruiz, D. A. Solís Casados, J. A. Pliego, C. M. Álvarez and E. S. de Andrés, Transformation of Methanol to Hydrocarbons (MTH): Comparison Between MeAPO-36 (Me = Zn, Co, Mg) and Modified ZSM-5 Catalysts, *Catal Letters*, 2023, **153**, 2493–2503.

18 D. E. Akporiaye, A. Andersen, I. M. Dahl, H. B. Mostad and R. Wendelbo, Synthesis and characterization of the magnesium silicoaluminophosphates MAPSO-43 and MAPSO-39, *J Phys Chem*, 1995, **99**, 14142–14148.

19 A. Nørlund Christensen, P. Norby and J. C. Hanson, Crystallization of microporous aluminophosphates and me2+-substituted aluminophosphates investigated by in situ synchrotron x-ray powder diffraction, *Microporous and Mesoporous Materials*, 1998, **20**, 349–354.

20 M. M. Carvalho, L. A. M. Ruotolo and R. Fernandez-Felisbino, Synthesis of aluminophosphate by the ionothermal method using factorial design, *Microporous and Mesoporous Materials*, 2013, **165**, 163–167.

21 Y. Xiu, Q. Gao, G.-D. Li, K.-X. Wang and J.-S. Chen, Preparation and Tunable Photoluminescence of Carbogenic Nanoparticles Confined in a Microporous Magnesium-Aluminophosphate, *Inorg Chem*, 2010, **49**, 5859–5867.

22 G. W. Noble, P. A. Wright, P. Lightfoot, R. E. Morris, K. J. Hudson, Å. Kvick and H. Graafsma, Microporous Magnesium Aluminophosphate STA-1: Synthesis with a Rationally Designed Template and Structure Elucidation by Microcrystal Diffraction, *Angewandte Chemie International Edition in English*, 1997, **36**, 81–83.

23 G. W. Noble, P. A. Wright and Å. Kvick, The templated synthesis and structure determination by synchrotron microcrystal diffraction of the novel small pore magnesium aluminophosphate STA-2 †, *Journal of the Chemical Society, Dalton Transactions*, 1997, 4485–4490.

24 P. A. Wright, R. H. Jones, S. Natarajan, R. G. Bell, J. Chen, M. B. Hursthouse and J. M. Thomas, Synthesis and structure of a novel large-pore microporous magnesium-containing aluminophosphate (DAF-1), *J Chem Soc Chem Commun*, 1993, 633.

25 Y. Wang, L. Shao, Y. Li, X. Li, J. Li, J. Yu and R. Xu, LEV-zeotype magnesium aluminophosphates with variable Mg/Al ratios, *Dalton Transactions*, 2012, **41**, 6855.

26 N. C. Masson and H. O. Pastore, Synthesis and characterization of tubular aluminophosphate mesoporous materials containing framework magnesium, *Microporous and Mesoporous Materials*, 2001, **44–45**, 173–183.

27 Y. Wang, Y. Li, Y. Yan, J. Xu, B. Guan, Q. Wang, J. Li and J. Yu, Luminescent carbon dots in a new magnesium aluminophosphate zeolite, *Chemical Communications*, 2013, **49**, 9006.

28 Y. Mu, Y. Wang, Y. Li, J. Li and J. Yu, Organotemplate-free synthesis of an open-framework magnesium aluminophosphate with proton conduction properties, *Chemical Communications*, 2015, **51**, 2149–2151.

29 Y. Wei, L. Zhang and Z. Tian, Isomorphous substitution induced ionothermal synthesis of magnesium aluminophosphate zeolites in fluoride-free media, *RSC Adv*, 2016, **6**, 61915–61919.

30 S. Gopalakrishnan, K. R. Viswanathan, S. V. Priya, J. H. Mabel, M. Palanichamy and V. Murugesan, Lewis acid metal ion-exchanged MAPO-5 molecular sieves for solvent free synthesis of coumarin derivative, *Catal Commun*, 2008, **10**, 23–28.

31 L. Feng, X. Qi, Z. Li, Y. Zhu and X. Li, Synthesis and Characterization of Magnesium-Substituted Aluminophosphate Molecular Sieves (MgAPO-11) and Their Kinetic Study of Catalytic Cracking of n-Hexane, *Chinese Journal of Catalysis*, 2009, **30**, 340–346.

32 P. Wang, H. Liu, C. Wang, G. Lv, D. Wang, H. Ma and Z. Tian, Direct synthesis of shaped MgAPO-11 molecular sieves and the catalytic performance in *n* -dodecane hydroisomerization, *RSC Adv*, 2021, **11**, 25364–25374.

33 U. Lohse, B. Parlitz, D. Müller, E. Schreier, R. Bertram and R. Fricke, MgAPO molecular sieves of CHA and AFI structure — Acidity and Mg ordering, *Microporous Materials*, 1997, **12**, 39–49.

34 S. K. Saha, S. B. Waghmode, H. Maekawa, R. Kawase, K. Komura, Y. Kubota and Y. Sugi, Magnesioaluminophosphate molecular sieves with ATS topology: Synthesis by dry-gel conversion method and catalytic properties in the isopropylation of biphenyl, *Microporous and Mesoporous Mater.*, 2005, **81**, 277–287.

35 D. B. Akolekar and S. K. Kaliaguine, Preparation and characterization of the magnesium aluminophosphate MAPO-39, *Zeolites*, 1994, **14**, 620–624.

36 A. K. Sinha, S. G. Hegde, N. E. Jacob and S. Sivasanker, Preparation and characterization of the silicoaluminophosphate SAPO-39, *Zeolites*, 1997, **18**, 350–355.

37 L. Washmon-Kriel and K. J. Balkus, Preparation and characterization of oriented MAPO-39 membranes, *Microporous and Mesoporous Materials*, 2000, **38**, 107–121.

38 F. Deng, Y. Yue, T. Xiao, Y. Du, C. Ye, L. An and H. Wang, Substitution of Aluminum in Aluminophosphate Molecular Sieve by Magnesium: A Combined NMR and XRD Study, *J Phys Chem*, 1995, **99**, 6029–6035.

39 J.-J. Li, Y. Guo, G.-D. Li, J.-S. Chen, C.-J. Li and Y.-C. Zou, Investigation into the role of MgO in the synthesis of MAPO-11 large single crystals, *Microporous and Mesoporous Materials*, 2005, **79**, 79–84.

40 K. Nakashiro, Y. Ono, S. Nakata and Y. Morimura, Characterizations of MAPO-36 molecular sieve by solid-state n.m.r., *Zeolites*, 1993, **13**, 561–564.

41 D. B. Akolekar, Novel, crystalline, large-pore magnesium aluminophosphate molecular sieve of type 50: Preparation, characterization, and structural stability, *Zeolites*, 1995, **15**, 583–590.

42 W.-L. Shea, R. B. Borade and A. Clearfield, Synthesis and properties of MgAPO-5, *Journal of the Chemical Society, Faraday Transactions*, 1993, **89**, 3143.

43 Y. Zhang, F. Zhang, G.-D. Li, J. Yang and J.-S. Chen, Carbon anode material formed from template molecules occluded in a magnesium-substituted aluminophosphate, *Mater Chem Phys*, 2009, **113**, 309–313.

44 Y. Chen, J. Zhai, X. Xu, I. Li, S. Ruan and Z. Tang, Synthesis of large MgAPO-11 single crystals in a F^- free system, *CrystEngComm*, 2014, **16**, 2984–2989.

45 D. B. Akolekar and S. Kaliaguine, MAPO-43 molecular sieve: Synthesis, characterization and thermal stability, *Microporous Materials*, 1994, **2**, 137–144.

46 P. Concepcion, J. M. Lopez Nieto and J. Perez-Pariente, Oxidative dehydrogenation of ethane on a magnesium-vanadium aluminophosphate (MgVAPO-5) catalyst, *Catal Letters*, 1994, **28**, 9–15.

47 P. Feng, X. Bu and G. D. Stucky, Amine-templated syntheses and crystal structures of zeolite rho analogs, *Microporous and Mesoporous Materials*, 1998, **23**, 315–322.

48 P. Feng, X. Bu and C.-S. Yang, Synthesis and single crystal structure of an AFX-type magnesium aluminophosphate, *Microporous and Mesoporous Materials*, 2001, **50**, 145–149.

49 K. O. Kongshaug, H. Fjellvåg and K. P. Lillerud, Synthesis and Crystal Structure of the Organically Templated Open Framework Magnesium Phosphate UiO-20 with DTF Topology, *Chemistry of Materials*, 2000, **12**, 1095–1099.

50 K. O. Kongshaug, H. Fjellvåg and K. P. Lillerud, Synthesis and characterization of the open-framework magnesium aluminophosphate UiO-28, *J Mater Chem*, 2001, **11**, 1242–1247.

51 Y.-X. Li, H.-T. Zhang, Y.-Z. Li, Y.-H. Xu and X.-Z. You, Hydrothermal synthesis and characterization of magnesium substituted aluminophosphate with unique intersecting 12-membered ring channels in three dimensions, *Microporous and Mesoporous Materials*, 2006, **97**, 1–8.

52 A. Oliden-Sánchez, R. Sola-Llano, J. Pérez-Pariente, L. Gómez-Hortigüela and V. Martínez-Martínez, Exploiting the photophysical features of DMAN template in ITQ-51 zeotype in the search for FRET energy transfer, *Physical Chemistry Chemical Physics*, 2024, **26**, 1225–1233.

53 F. Dawaymeh, D. A. Gaber, S. A. Gaber, K. S. K. Reddy, G. Basina, I. Ismail, A. Subrati, O. Ismail, G. N. Karanikolos and Y. F. Al Wahedi, Water adsorption behavior of Mg and Fe substituted microporous AlPO4-5, *J. Water Process Eng.*, 2022, **49**, 103079.

54 S. Vishnu Priya, J. Herbert Mabel, S. Gopalakrishnan, M. Palanichamy and V. Murugesan, Lewis acid metal ion-exchanged MAPO-36 molecular sieve: Characterisation and catalytic activity, *J Mol Catal A Chem*, 2008, **290**, 60–66.

55 J. Zhang, H. Wu, A. Zhao, X. Bai, O. V. Kikhtyanin, W. Wu, L. Xiao, X. Su and R. Zhang, Synthesis of MgAPO-31 nanocrystals via different heating methods and their catalytic performance in the hydroisomerization of n-decane, *Journal of Porous Materials*, 2017, **24**, 437–442.

56 D. B. Akolekar, Structural stability and surface, acidic, and catalytic properties of magnesium aluminophosphate of type 36: Influence of ratios and of thermal and hydrothermal treatments, *J Catal*, 1994, **146**, 62–68.

57 P. A. Wright, C. Sayag, F. Rey, D. W. Lewis, J. D. Gale, S. Natarajan and J. M. Thomas, Synthesis, characterisation and catalytic performance of the solid acid DAF-1, *Journal of the Chemical Society, Faraday Transactions*, 1995, **91**, 3537.

58 S. K. Saha, S. B. Waghmode, H. Maekawa, K. Komura, Y. Kubota, Y. Sugi, Y. Oumi and T. Sano, Synthesis of aluminophosphate molecular sieves with AFI topology substituted by alkaline earth metal and their application to solid acid catalysis, *Microporous and Mesoporous Mater.*, 2005, **81**, 289–303.

59 V. R. Vijayaraghavan and K. J. A. Raj, Ethylation of benzene with ethanol over substituted large pore aluminophosphate-based molecular sieves, *J Mol Catal A Chem*, 2004, **207**, 41–50.

60 G. Lischke, B. Parlitz, U. Lohse, E. Schreier and R. Fricke, Acidity and catalytic properties of MeAPO-5 molecular sieves, *Appl Catal A Gen*, 1998, **166**, 351–361.

61 T. Cordero-Lanzac, I. Capel Berdiell, A. Airi, S.-H. Chung, J. L. Mancuso, E. A. Redekop, C. Fabris, L. Figueroa-Quintero, J. C. Navarro de Miguel, J. Narciso, E. V. Ramos-Fernandez, S. Svelle, V. Van Speybroeck, J. Ruiz-Martínez, S. Bordiga and U. Olsbye, Transitioning from Methanol to Olefins (MTO) toward a Tandem CO_2 Hydrogenation Process: On the Role and Fate of Heteroatoms (Mg, Si) in MAPO-18 Zeotypes, *JACS Au*, 2024, **4**, 744–759.

62 F. Dawaymeh, D. A. Gaber, S. A. Gaber, K. S. K. Reddy, G. Basina, I. Ismail, A. Subrati, O. Ismail, G. N. Karanikolos and Y. F. Al Wahedi, Water adsorption behavior of Mg and Fe substituted microporous AlPO4-5, *J. Water Process Eng.*, 2022, **49**, 103079.

63 A. A. Coelho, TOPAS and TOPAS-Academic : an optimization program integrating computer algebra and crystallographic objects written in C++, *J Appl Crystallogr*, 2018, **51**, 210–218.

64 A. A. Coelho, J. Evans, I. Evans, A. Kern and S. Parsons, The TOPAS symbolic computation system, *Powder Diffr*, 2011, **26**, S22–S25.

65 Robert. E. Dinnebier, A. Leineweber and John. S. O. Evans, *Rietveld Refinement. Practical Powder Diffraction Pattern Analysis using TOPAS*, De Gruyter, 2018.

66 G. J. Klap, H. van Koningsveld, H. Graafsma and A. M. M. Schreurs, Absolute configuration and domain structure of AlPO4-5 studied by single crystal X-ray diffraction, *Microporous and Mesoporous Materials*, 2000, **38**, 403–412.

67 S. Qiu, W. Pang, H. Kessler and J.-L. Guth, Synthesis and structure of the [AlPO4]12 Pr4NF molecular sieve with AFI structure, *Zeolites*, 1989, **9**, 440–444.

68 M. Calligaris, G. Nardin and L. Randaccio, Cation site location in hydrated chabazites. Crystal structure of potassium- and silver- exchanged chabazites, *Zeolites*, 1983, **3**, 205–208.
